# Supplementary material for: The two kinds of free energy and the Bayesian revolution
Source: PLoS Comput Biol. 2020 Dec 3;16(12):e1008420. doi: 10.1371/journal.pcbi.1008420 (PMC7714236; doi:10.1371/journal.pcbi.1008420)
Supplement: S1 Appendix — We derive update equations of Q-value and direct Active Inference for the example in Section 5.2 under mean-field and Bethe approximations. (PDF) [file pcbi.1008420.s001.pdf]

The two kinds of free energy and the Bayesian revolution

Supporting Information S1 Appendix

Sebastian Gottwald, Daniel A. Braun

Derivation of exemplary update equations

A. Q-value Active Inference

In the simple example of Section 5.2 under the partial mean-field assumption (23), and in the case when the desired distribution $p_{\rm des}$ is combined with the generative model p_0 via the value function Q as shown in Equation (24), i.e. if $\phi \propto p_0(x, X', \mathbf{S}, A)\, e^{Q(A)}$, then the full free energy $F(q\|\phi)$ can be written as

$$\begin{aligned} F(q\|\phi) &= F(q(\mathbf{S}|A)q(A)\|p_0(x|S)p_0(\mathbf{S}|A)p_0(A)e^{Q(A)}) \\ &= \Big\langle \underbrace{F\big(q(\mathbf{S}|A)\|p_0(x|S)p_0(\mathbf{S}|A)\big)}_{=:\, F_{\mathbf{S}}(A)} - Q(A) \Big\rangle_{q(A)} + D_{\rm KL}(q(A)\|p_0(A))\,, \end{aligned} \tag{A1}$$

where, explicitly

$$F_{\mathbf{S}}(A) - Q(A) = \left\langle \log \frac{q(S) \quad q(S'|A) \qquad \sum_{s'} p_0(X'|s')q(s'|A)}{p_0(x|S)p_0(S)\, p_0(S'|S,A) \quad p_{\rm des}(X')p_0(X'|S')} \right\rangle_{q(X',\mathbf{S}|A)} . \tag{A2}$$

Thus, optimizing (A1) over $q(A)$, while keeping $q(\mathbf{S}|A)$ fixed, results in a Boltzmann distribution with prior $p_0(A)$ and energy $F_{\mathbf{S}}(A) - Q(A)$. When optimizing $F(q\|\phi)$ with respect to $q(S)$ while keeping $q(S'|A)$ and $q(A)$ fixed, we have

$$\begin{aligned} q^*(S) &= \underset{q(S)}{\operatorname{argmax}}\ F(q\|\phi) = \underset{q(S)}{\operatorname{argmax}}\ \langle F_{\mathbf{S}}(A)\rangle_{q(A)} \\ &= \underset{q(S)}{\operatorname{argmax}}\ \underbrace{\left\langle \log \frac{q(S)}{p_0(x|S)p_0(S)e^{\langle T\rangle_{q(S'|A)q(A)}}} \right\rangle_{q(S)}}_{F\left(q(S)\big\|p_0(x|S)p_0(S)e^{\langle T\rangle}\right)}\,, \end{aligned} \tag{A3}$$

where $T := \log p_0(S'|S, A)$ is shorthand for the log-transition probability. Hence, from (A3) we can read off the solution $q^*(S)$ in virtue of the general optimum (14) of variational free energy. While here it was enough to optimize $\langle F_{\mathbf{S}}\rangle_q$, because it contains the only dependencies of $F(q\|\phi)$ on $q(S)$, this is not the case for $q(S'|A)$, since also Q depends on $q(S'|A)$. Thus, when optimizing (A1) over $q(S'|A)$ while keeping $q(A)$ and $q(S)$ fixed, one has to optimize $\langle F_{\mathbf{S}} - Q\rangle$ which does not take the form of a free energy in $q(S'|A)$ due to the functional dependency of $q(X'|A) = \sum_{s'} p_0(X'|s')q(s'|A)$ on $q(S'|A)$ that appears in (A2). However, this type of dependency is largely ignored in the Active Inference literature (as for example noted in the appendix of [1]), since the optimization with respect to $q(S'|A)$ would not have a closed-form solution otherwise.

Once this term is ignored, then the objective for $q(S'|A)$ takes a very simple form,

$$q^*(S'|A) = \underset{q(S'|A)}{\operatorname{argmax}}\ F(q\|\phi) \approx \underset{q(S'|A)}{\operatorname{argmax}} \left\langle \log \frac{q(S'|A)}{e^{\langle T\rangle_{q(S)}}} \right\rangle_{q(S'|A)}\,, \tag{A4}$$

from which we can again read off the resulting update equation. In total, from (A1),(A3), and (A4) we obtain the set of equations

$$q^*(S) = \tfrac{1}{\mathcal{Z}}\, p_0(x|S)p_0(S)e^{\langle T\rangle_{q(S'|A)q(A)}} \tag{A5a}$$

$$q^*(S'|A) \approx \tfrac{1}{\mathcal{Z}(A)}\, e^{\langle T\rangle_{q(S)}} \tag{A5b}$$

$$q^*(A) = \tfrac{1}{\mathcal{Z}}\, p_0(A)e^{-F_{\mathbf{S}}(A)+Q(A)}, \tag{A5c}$$

where $\mathcal{Z}$ denotes the respective normalization constants and $T = \log p_0(S'|S,A)$.

It is important to note, however, that update equations in Active Inference resulting from a mean-field assumption (even if it is a partial mean-field assumption such as (23)) should be taken with care, since—as is demonstrated in the grid world simulations in S2 Notebook—even in very simple situations the resulting agents fail to correctly plan actions that lead to desired states.

B. Direct Active Inference (variational Control as Inference)—mean-field assumption

Here, we derive the update equations resulting from the minimization of the variational free energy for the reference defined in Equation (25), i.e. a variational formulation of Control as inference [2], under the mean-field assumption (23). We start by writing the variational free energy $F(q\|\phi)$ in a form analogous to (A1), where now ϕ is given by $p_0(X'|S')p_0(x,\mathbf{S}|A)p_0(A)$,

$$F(q\|\phi) = \Big\langle \underbrace{F(q(\mathbf{S}|A)\|p_0(x,\mathbf{S}|A))}_{=F_{\mathbf{S}}(A)} - G(A) \Big\rangle_{q(A)} + D_{\mathrm{KL}}(q(A)\|p_0(A)),$$

where

$$G(A) := \Big\langle \underbrace{\langle \log p_{\mathrm{des}}(X')\rangle_{p_0(X'|S')}}_{=:g(S')} \Big\rangle_{q(S'|A)}.$$

Note that, compared to Q-value Active Inference, here we do not have to make any additional approximations, because G only depends linearly on $q(S'|A)$.

Similarly to above, when optimizing with respect to $q(A)$ while keeping $q(S)$ and $q(S'|A)$ fixed, we obtain that $q^*(A)$ is a Boltzmann distribution with energy $F_{\mathbf{S}} - G$ and prior $p_0(A)$. Optimizing $q(S)$ while keeping $q(A)$ and $q(S'|A)$ constant has the same result as shown in (A5a) because as before the only dependencies on $q(S)$ are in $F_{\mathbf{S}}$. Finally, in order to read off the solution of the optimization with respect to $q(S'|A)$ while keeping $q(S)$ and $q(A)$ constant, we can rewrite $F_{\mathbf{S}} - G$ as follows

$$\begin{aligned} q^*(S'|A) &= \underset{q(S'|A)}{\operatorname{argmax}}\ F(q\|\phi) = \underset{q(S'|A)}{\operatorname{argmax}}\big(F_{\mathbf{S}}(A) - G(A)\big) \\ &= \underset{q(S'|A)}{\operatorname{argmax}} \Big\langle \log \frac{q(S'|A)}{e^{\langle T\rangle_{q(S)} + g(S')}} \Big\rangle_{q(S'|A)}, \end{aligned}$$

so that in total we obtain the set of equations

$$q^*(S) = \tfrac{1}{\mathcal{Z}}\, p_0(x|S)p_0(S)e^{\langle T\rangle_{q(S'|A)q(A)}} \tag{A6a}$$

$$q^*(S'|A) = \tfrac{1}{\mathcal{Z}(A)}\, e^{\langle T\rangle_{q(S)} + g(S')} \tag{A6b}$$

$$q^*(A) = \tfrac{1}{\mathcal{Z}}\, p_0(A)e^{-F_{\mathbf{S}}(A)+G(A)}, \tag{A6c}$$

where $\mathcal{Z}$ denotes the normalization constants, and again $T = \log p_0(S'|S,A)$.

It is noteworthy that recently another free energy approach similar to Active Inference has been introduced that does not make use of variational free energy, but of a different functional termed *generalized free energy* [3]. Despite of the different functional form, this version uses a reference function that is similar to the direct Active Inference approach [4], where the desired distribution is also multiplied directy to the generative model but with a renormalization that results in a modified generative model over observations, states, and actions. Using this renormalized reference in a variational free energy approach would result in trivial inference reproducing the fixed prior $p_0(A)$, corresponding to Bayes' conditioning the modified generative model on the past analogous to perceptual Bayesian inference, e.g. $p(A|X) = p_0(A)$ in the case of the one-step example. In contrast, the minimization of the free energy functional used in [3] does not correspond to a Bayesian inference process, which is why we do not further discuss it here.

C. Direct Active Inference (variational Control as Inference)—Bethe assumption

Here, we derive the update equations resulting from the minimization of the variational free energy for the reference (25) under a Bethe approximation, which therefore is a more precise variational formulation of Control as Inference as the mean-field approximation of the previous section. In fact, it turns out that such equations are equivalent to Belief propagation [5], a well-known inference method that produces exact marginals in tree-like graphs [6], such as the probabilistic models considered in the article and in the Active Inference literature.

Analogous to the previous section, without any specific restrictions on q we can write the total free energy for the one-step example from Section 5.2 with the reference (25a) as

$$F(q\|\phi) = \underbrace{\left\langle \log \frac{q(X', S, S'|A)}{p_0(R\!=1, X\!=x, X', S, S'|A)} \right\rangle_{q(X',S',S|A)q(A)}}_{=:\, \langle F(A)\rangle_{q(A)}} + D_{\mathrm{KL}}(q(A)\|p_0(A))$$

from which it immediately follows that minimizing with respect to $q(A)$, while considering $q(X', S, S'|A)$ constant, results in a Boltzmann distribution with energy $F(A)$ and prior $p_0(A)$. $F(A)$ is the variational free energy of $q(X', S, S'|A)$ with respect to the reference

$$p_0(R\!=1, X\!=x, X', S, S'|A) = \underbrace{p_0(x|S)p_0(S)}_{=:f_1(S)}\underbrace{p_0(S'|S,A)}_{=:f_2(S,S')}\underbrace{p_0(X'|S')}_{=:f_3(S',X')}\underbrace{p_{\mathrm{des}}(X')}_{=:f_4(X')}\,.$$

Thus, minimizing $F(A)$ with respect to $q(X', S, S'|A)$ without any restrictions or simplifications results in the exact Bayes' posterior

$$p(X', S, S'|A, R = 1, X = x) = \frac{1}{\mathcal{Z}(A)}\, f_1(S)\, f_2(S, S')\, f_3(S', X')\, f_4(X')\,,$$

where $\mathcal{Z}(A)$ denotes the corresponding normalization constant. The problem that we want to solve is to find an approximation to this Bayes' posterior that is more precise than the mean-field approximation of the previous section but requires less involved computations than the determination of $\mathcal{Z}(A)$. While one attempt is to partition the full graph into smaller graphs and apply a naive mean-field approximation inside of each subgraph, known as a *structured* mean-field approximation [7], the Bethe approximation follows a slightly different approach. It is the simplest version of the *cluster variation methods* often attributed to Kikuchi [8], a family of region-based free energy

approximations [9], where one keeps beliefs over different sections of the factor graph. Specifically, in the Bethe assumption, the regions consist of each factor and its neighbouring nodes, which can also be seen as allowing pair-wise interactions. Following the systematic treatment in [9], the Bethe approximation for our example consists of seven belief functions, one for each factor, $b_1, \ldots, b_4$, and one for each variable, b_S, $b_{S'}$, and $b_{X'}$,

$$q(S, S', X'|A) = \frac{b_1(S) b_2(S, S') b_3(S', X') b_4(X')}{b_S(S) b_{S'}(S') b_{X'}(X')} \tag{A7}$$

where the marginals of the factor beliefs are required to be consistent with the single-variable beliefs. Thus, the variational free energy $F(A)$ can be written as

$$F(A) = \sum_{k=1}^{4} \left\langle \log \frac{b_k}{f_k} \right\rangle_{b_k} - \sum_{Y \in \{S, S', X'\}} \langle \log b_Y \rangle_{b_Y}$$

which has to be minimized under the consistency and normalization contraints, leading to the Lagrangian

$$\begin{aligned} F(A) &+ \sum_s \lambda_1(s) \big(b_S(s) - b_1(s)\big) \\ &+ \sum_s \lambda_{2S}(s) \left(b_S(s) - \sum_{s'} b_2(s, s') \right) + \sum_{s'} \lambda_{2S'}(s') \left(b_{S'}(s') - \sum_s b_2(s, s') \right) \\ &+ \sum_{s'} \lambda_{3S'}(s') \left(b_{S'}(s') - \sum_{x'} b_3(s', x') \right) + \sum_{x'} \lambda_{3X'}(x') \left(b_{X'}(x') - \sum_{s'} b_3(s', x') \right) \\ &+ \sum_{x'} \lambda_4(x') \big(b_{X'}(x') - b_4(x')\big) \\ &+ \sum_{k=1}^{4} \gamma_k \left(\sum b_k - 1 \right) + \sum_{Y \in \{S, S', X'\}} \gamma_Y \left(\sum b_Y - 1 \right) \end{aligned}$$

where the Lagrange multipliers for the consistency constraints are denoted by λ and the Lagrange multipliers for the normalization constraints by γ. The equations for the beliefs at the stationary points (zeroes of the derivatives of the Lagrangian) are

$$\begin{aligned} b_1(s) &\propto f_1(s)\, e^{\lambda_1(s)}\,, & b_S(s) &\propto e^{\lambda_1(s)}\, e^{\lambda_{2S}(s)}\,, \\ b_2(s, s') &\propto f_2(s, s')\, e^{\lambda_{2S}(s)}\, e^{\lambda_{2S'}(s')}\,, & b_{S'}(s') &\propto e^{\lambda_{2S'}(s')}\, e^{\lambda_{3S'}(s')}\,, \\ b_3(s', x') &\propto f_3(s', x')\, e^{\lambda_{3S'}(s')}\, e^{\lambda_{3X'}(x')}\,, & b_{X'}(x') &\propto e^{\lambda_{3X'}(x')}\, e^{\lambda_4(x')}\,, \\ b_4(x') &\propto f_4(x')\, e^{\lambda_4(x')}\,, \end{aligned}$$

where the proportionality sign $\propto$ means that the left-hand side results from normalizing the right hand-side to obtain a probability distribution. By writing $m_l := e^{\lambda_l}$ for all $l \in \{1, 2S, 2S', 3S', 3X', 4\}$, we obtain from the stationarity conditions and the consistency constraints

$$m_{2S}(s) \propto f_1(s) \tag{A8a}$$

$$m_1(s) \propto \sum\nolimits_{s'} f_2(s, s')\, m_{2S'}(s') \tag{A8b}$$

$$m_{3S'}(s') \propto \sum\nolimits_{s} f_2(s, s')\, m_{2S}(s) \tag{A8c}$$

$$m_{2S'}(s') \propto \sum\nolimits_{x'} f_3(s', x')\, m_{3X'}(x') \tag{A8d}$$

$$m_4(x') \propto \sum\nolimits_{s'} f_3(s', x')\, m_{3S'}(s') \tag{A8e}$$

$$m_{3X'}(x') \propto f_4(x')\,. \tag{A8f}$$

The update equations for the beliefs in (A7) can be obtained by iterating the equations in (A8) and using the stationarity conditions that express the beliefs in terms of the m_l. Note that the quantities denoted by m_l are usually interpreted as local messages that are sent between the nodes and factors of the underlying graphical model [9], e.g. $m_{3S'}$ is considered a message sent from node S' to factor 3, which can be used to determine the message m_4 from factor 3 to node X' by weighing with f_3 and summing over S', etc. By this identification, variational inference under the Bethe approximation is equivalent to belief propagation. While in (A8) there is at most one message that is multiplied to the factor f_k before the sum is taken, in more complex factor graphs, where more than 2 nodes are connected to a factor, the messages coming in to a factor from the neighboring nodes are multiplied before they are summed to calculate the outgoing message, which is why this type of message-passing is also known as the *sum-product* algorithm.

References

1. Friston KJ, Rigoli F, Ognibene D, Mathys C, Fitzgerald T, Pezzulo G. Active inference and epistemic value. Cognitive Neuroscience. 2015;6(4):187–214.

2. Toussaint M, Storkey A. Probabilistic Inference for Solving Discrete and Continuous State Markov Decision Processes. In: Proceedings of the 23rd International Conference on Machine Learning. ICML '06. New York, NY, USA: Association for Computing Machinery; 2006. p. 945–952.

3. Parr T, Friston KJ. Generalised free energy and active inference. Biological Cybernetics. 2019;doi:10.1007/s00422-019-00805-w.

4. Schwöbel S, Kiebel S, Marković D. Active Inference, Belief Propagation, and the Bethe Approximation. Neural Computation. 2018;30(9):2530–2567.

5. Yedidia JS, Freeman WT, Weiss Y. Generalized Belief Propagation. In: Leen TK, Dietterich TG, Tresp V, editors. Advances in Neural Information Processing Systems 13. MIT Press; 2001. p. 689–695.

6. Pearl J. Belief Updating by Network Propagation. In: Pearl J, editor. Probabilistic Reasoning in Intelligent Systems. San Francisco (CA): Morgan Kaufmann; 1988. p. 143–237.

7. Saul LK, Jordan MI. Exploiting Tractable Substructures in Intractable Networks. In: Touretzky DS, Mozer MC, Hasselmo ME, editors. Advances in Neural Information Processing Systems 8. MIT Press; 1996. p. 486–492.

8. Kikuchi R. A Theory of Cooperative Phenomena. Physical Review. 1951;81(6):988–1003. doi:10.1103/physrev.81.988.

9. Yedidia JS, Freeman WT, Weiss Y. Constructing free-energy approximations and generalized belief propagation algorithms. IEEE Transactions on Information Theory. 2005;51(7):2282–2312. doi:10.1109/TIT.2005.850085.
