# Supplementary material for: The two kinds of free energy and the Bayesian revolution
Source: PLoS Comput Biol. 2020 Dec 3;16(12):e1008420. doi: 10.1371/journal.pcbi.1008420 (PMC7714236; doi:10.1371/journal.pcbi.1008420)
Supplement: S2 Appendix — We give additional details on the example shown in Fig 8 that illustrates the effects of assuming a particular desired distribution over three outcomes under Jeffrey conditionalization, Control as Inference, expected utility optimization, and Active Inference. (PDF) [file pcbi.1008420.s002.pdf]

The two kinds of free energy and the Bayesian revolution
Supporting Information S2 Appendix

Sebastian Gottwald, Daniel A. Braun

Uncertain and deterministic options (example in Fig 8)

Here, we are giving additional details on Fig 8 in the article. We consider the simple example of three possible observations, x_1, x_2, x_3, a desired distribution $p_{\mathrm{des}} = (1/3, 1/6, 1/2)$, two actions a with predictive distributions $p(X'|A{=}1) = (1, 0, 0)$ and $p(X'|A{=}2) = (0, 1/2, 1/2)$, and a constant prior $p_0(A) = (1/2, 1/2)$. We can consider $p(X'|A)$ as a result of marginalizing the generative model $p_0(X', S', A) = p_0(X'|S')p_0(S'|A)p_0(A)$ with state distributions $p(S'|A{=}1) = (1, 0, 0)$ and $p(S'|A{=}2) = (0, 1/2, 1/2)$ and an emission probability $p_0(X'|S')$ that is chosen such that the given $p(X'|A)$ equals

$$p(X'|A) = \sum_{s'} p_0(X'|s')p_0(s'|A).$$

Suitable emission probabilities have for example the form $p_0(X'{=}x_i|S'{=}s_j) = M_{ij}(t)$, where

$$M(t) = \begin{pmatrix} 1 & 0 & 0 \\ 0 & t & 1{-}t \\ 0 & 1{-}t & t \end{pmatrix}$$

for all $t \in [0, 1]$. Note that the resulting average entropies $\langle H(p_0(S'|A{=}2))\rangle_{p_0(S'|A=2)}$ are in the range $[0, 1]$ bit for $A = 2$ (always zero for $A = 1$), where the extreme values are assumed at $t \in \{0, 1\}$ (0 bit) and $t = 1/2$ (1 bit).

Furthermore, for the application of Active Inference in Fig 8, we have considered an exact version of the value function, $Q = Q_{\mathrm{exact}}$, where the trial distribution $q(S'|A)$ is replaced by the exact predictive distribution $p_0(S'|A)$. In this "exact" interpretation, the corresponding action distributions $p(A) \propto p_0(A)e^{Q_{\mathrm{exact}}(A)}$ could then be viewed as defining the ideal behaviour that is approximated by the variational free energy minimization. In the Active Inference literature, $p(A) \propto p_0(A)e^{Q(A)}$ is considered a "prior", because it is viewed as part of the generative model and thus is part the input to the variational inference process. However, by considering Q an approximation of Q_{exact} these distributions can be viewed as defining the ideal behavior that is approximated by the trial distributions during free energy minimization and are therefore more in line with the "posteriors" in other decision-making models (even though the value function $Q(A)$—and therefore $p(A)$—is presupposed, in constrast to being the result of some principle).
