# Supplementary material for: The two kinds of free energy and the Bayesian revolution
Source: PLoS Comput Biol. 2020 Dec 3;16(12):e1008420. doi: 10.1371/journal.pcbi.1008420 (PMC7714236; doi:10.1371/journal.pcbi.1008420)
Supplement: S3 Appendix — Explanation of the relation between free energy minimization, free energy as a bound on surprise, and surprise minimization. (PDF) [file pcbi.1008420.s003.pdf]

The two kinds of free energy and the Bayesian revolution
Supporting Information S3 Appendix

Sebastian Gottwald, Daniel A. Braun

Surprise minimization

The (*informational*) *surprise* or *surprisal* of a given element x with respect to a probability distribution $p_0(X)$ is defined as $S_0 := -\log p_0(x)$, i.e. it is simply a strictly decreasing function of probability such that outcomes x with low probability have high surprise and outcomes x with high probability have low surprise. A common statement found in the literature [1] is that variational free energy is an upper bound on surprise and thus minimizing free energy also minimizes surprise. This idea originates from the special case of greedy inference with latent variables, where, for fixed data x, the goal is to maximize the likelihood $p_\theta(x) = \sum_z p_\theta(x,z)$ with respect to a parameter θ. If the marginalization over the latent variable Z is too hard to carry out directly, then one might take advantage of the bound

$$F(q(Z)\|p_\theta(x,Z)) \geq -\log p_\theta(x) =: S_\theta, \tag{A1}$$

i.e. that the variational free energy of $q(Z)$ is an upper bound on the surprise S_θ, which might therefore be reduced by minimizing its upper bound *with respect to* θ as a proxy. In the variational Bayes' approach to the above inference problem, where θ is treated as a random variable Θ, minimization with respect to θ is replaced by the minimization with respect to $q(\Theta)$. In this case, the analogous bound to (A1) is

$$F(q(Z|\Theta)q(\Theta)\|p_0(x,Z,\Theta)) \geq -\log \sum_z e^{\langle \log p_0(x,z,\Theta)\rangle_{q(\Theta)}},$$

where the right-hand side is the minimum of the left-hand side with respect to $q(Z|\Theta)$. In this sense, variational free energy is generally not a bound on the surprise S_Θ anymore, but on a log-sum-exp version of it instead. Nonetheless, also in this Bayesian approach, variational free energy is an upper bound on the surprise S_0,

$$F(q(Z|\theta)q(\theta)\|p_0(x,Z,\Theta)) \geq -\log p_0(x) = S_0, \tag{A2}$$

where the right-hand side is the minimum of the left-hand side with respect to both $q(Z|\theta)$ and $q(\theta)$. However, in contrast to (A1), there is no variable left in S_0 over which one could minimize. Therefore, saying that minimizing free energy also minimizes surprise [1], is generally only true in the sense that minimizing free energy minimizes an upper bound on surprise, however surprise itself is not minimized. Instead, the important fact about (A2) is that equality is achieved by the Bayes' posteriors $q(Z|\Theta) = p_0(Z|\Theta, x)$ and $q(\Theta) = p_0(\Theta|x)$ as discussed in Section 3.2.1.

References

1. Parr T, Friston KJ. Working memory, attention, and salience in active inference. Scientific reports. 2017;7(1):14678–14678. doi:10.1038/s41598-017-15249-0.
