# Supplementary material for: The two kinds of free energy and the Bayesian revolution
Source: PLoS Comput Biol. 2020 Dec 3;16(12):e1008420. doi: 10.1371/journal.pcbi.1008420 (PMC7714236; doi:10.1371/journal.pcbi.1008420)
Supplement: S4 Appendix — Discussion of how model and state variables can be separated in variational Bayesian inference which motivates the optimization scheme chosen by Active Inference. (PDF) [file pcbi.1008420.s004.pdf]

The two kinds of free energy and the Bayesian revolution
Supporting Information S4 Appendix

Sebastian Gottwald, Daniel A. Braun

Separation of model and state variables

In Q-value Active Inference, action and perception do not optimize the same variational free energy but two different free energy expressions. This is motivated from the separation of model variables M and state variables in standard variational Bayesian inference, where the full free energy can be split up into a sum of a state free energy F_M averaged over models M and a KL term that is independent of state distributions. Optimizing the full free energy can then be done separately by alternatingly doing perceptual inference by optimizing F_M for each model M and optimizing the full free energy to find the model distribution $q(M)$. In Active Inference, where actions A might be thought of analogous to models M in Bayesian inference, the full free energy is analogously split up into a sum of a state free energy F_A averaged over actions A and a KL term which—in contrast to standard Bayesian inference—*does* depend on state distributions. However, Active Inference essentially ignores this extra q-dependency by following the analogous optimization scheme to Bayesian inference: one alternatingly optimizes F_A with respect to state distributions and then the full free energy with respect to the action distributions $q(A)$. In particular, this separation into state and action free energies is not a consequence of optimizing the full variational free energy, but a deliberate choice made by Active Inference.

In the following, we discuss in more detail how this separation follows from optimizing the full free energy in standard Bayesian inference and highlight how Q-value Active Inference adopts the same optimization scheme but by giving up the optimization of a single variational free energy.

A. Bayesian inference

Consider the case of multiple probabilistic models $p_m(X, Z)$ that are indexed by a label m, where each p_m describes a different probabilistic relationship between data X and hidden states Z. Given data $X = x$, one could find the best m by selecting the model with the largest marginal likelihood $p_m(x) = \sum_z p_m(x, z)$. A popular method to accomplish this is the basic EM algorithm [1], where m is optimized greedily while Z is inferred using Bayesian inference for a given m (either exact or approximate). In a purely Bayesian treatment, one also assumes a prior distribution over models $p_0(M)$, so that the full joint over data X, hidden states Z, and models M becomes

$$p_0(X, Z, M) \;:=\; p_M(X, Z)\, p_0(M) \;=:\; p_0(X, Z|M)\, p_0(M)\,.$$

The Bayes' posterior $p(M|X)$ can then simply be determined from the Bayes' posterior $p(Z, M|X)$ through marginalization over Z. As discussed in the article (Section 3.2), if direct Bayesian inference is infeasable then a variational formulation might be useful,

where trial distributions $q(Z, M)$ over the unknown variables M and Z are fitted to the reference $\phi(Z, M) := p_0(x, Z, M)$ by minimizing the variational free energy

$$F(q\|\phi) = \left\langle \log \frac{q(Z,M)}{p_0(x,Z,M)} \right\rangle_{q(Z,M)} .$$

By writing q and p_0 in their factorized forms

$$q(Z, M) = q(Z|M)q(M), \quad p_0(X, Z, M) = p_0(X, Z|M)p_0(M) ,$$

the variational free energy can be decomposed as

$$\begin{aligned} F(q\|\phi) &= \left\langle \log \frac{q(Z|M)q(M)}{p_0(x,Z|M)p_0(M)} \right\rangle_q \\ &= \Big\langle \underbrace{\left\langle \log \frac{q(Z|M)}{p_0(x,Z|M)} \right\rangle_{q(Z|M)}}_{=:F_M} \Big\rangle_{q(M)} + \left\langle \log \frac{q(M)}{p_0(M)} \right\rangle_{q(M)} \\ &= \langle F_M \rangle_{q(M)} + D_{\mathrm{KL}}(q(M)\|p_0(M)) . \end{aligned} \tag{A1}$$

Notably, the minimization of F with respect to q splits up into the minimization of the free energy over states

$$F_M = F\big(q(Z|M)\big\|p_0(x, Z|M)\big) \tag{A2}$$

with respect to $q(Z|M)$, and the minimization of (A1) with respect to $q(M)$. In particular, the inference over models and states, (M, Z), separates into inference over hidden states for each model, which determines F_M for each M, and inference over M.

B. Active Inference

In Q-value Active Inference, action selection is treated similarly to model selection in Bayesian inference discussed in the previous section. However, the KL term in (A1) also depends on trial distributions over states, which means that a separation into action and state variables analogous to the separation in model selection is not possible when considering the problem of action and perception as the minimization of a single free energy functional, which is usually the conceptual starting point in the Active Inference literature [2, 3].

More precisely, as discussed in Section 5, the reference function ϕ that enters the variational free energy in Q-value Active Inference is constructed from a given probabilistic model p_0 and a value function Q by replacing the fixed prior $p_0(A)$ over actions with the modified distribution $\tilde{p}_0(A) := \frac{1}{Z}\, p_0(A)e^{Q(A)}$. As can be seen exemplarily in the one-step case in Equation (26), the value function Q depends on trial distributions q over hidden states and therefore $\tilde{p}_0(A)$ depends on q as well. Despite this dependency, the total free energy $F(q\|\phi)$ can still be written as

$$F(q\|\phi) = \left\langle \log \frac{q(X', \mathbf{S}|A)q(A)}{p_0(x, X', \mathbf{S}|A)\tilde{p}_0(A)} \right\rangle_q = \langle F_A \rangle_{q(A)} + D_{\mathrm{KL}}\big(q(A)\|\tilde{p}_0(A)\big) \tag{A3}$$

with

$$F_A := F\big(q(X', \mathbf{S}|A)\big\|p_0(x, X', \mathbf{S}|A)\big) . \tag{A4}$$

in the case of the one-step example of Section 5.2. Equations (A3) and (A4) are analogous to Equations (A1) and (A2), respectively. However, when optimizing the full free energy $F(q\|\phi)$ with respect to the factor $q(X', \mathbf{S}|A)$, one would have to consider both terms in the decomposition (A3), since, unlike $p_0(M)$ in the previous section, here

$\tilde{p}_0(A)$ does depend on trial distributions over states (the factor $q(S'|A)$). It should be noted that this dependency is non-linear and non-local, and therefore a closed-form solution cannot be derived (cf. (*ii*) in Section 5.3).

In Active Inference, this complication is avoided by simply ignoring the q-dependency of Q when deriving the update equations, or, put differently, one optimizes two different free energies for perception and action: one first optimizes F_A with respect to state distributions for each action A and then one optimizes the full free energy (A3) with respect to $q(A)$. This is in analogy to Bayesian model selection of the previous section, where this separation was a consequence of the minimization of the full free energy. However, here, due to the extra dependency of $\tilde{p}_0(A)$ on q, it is not a consequence but a choice made by Active Inference. This means that one no longer does variational inference over the combined set of states and actions, but variational inference over states with free energy F_A and variational inference over actions with free energy (A3). In particular, there is not a single free energy that is optimized by both perception and action, but two different ones.

References

1. Dempster AP, Laird NM, Rubin DB. Maximum Likelihood from Incomplete Data via the EM Algorithm. Journal of the Royal Statistical Society Series B (Methodological). 1977;39(1):1–38.

2. Friston KJ. The free-energy principle: a unified brain theory? Nature Reviews Neuroscience. 2010;11:127–138. doi:10.1038/nrn2787.

3. Friston K. Does predictive coding have a future? Nature Neuroscience. 2018;21(8):1019–1021. doi:10.1038/s41593-018-0200-7.
