# Supplementary material for: The two kinds of free energy and the Bayesian revolution
Source: PLoS Comput Biol. 2020 Dec 3;16(12):e1008420. doi: 10.1371/journal.pcbi.1008420 (PMC7714236; doi:10.1371/journal.pcbi.1008420)
Supplement: S1 Notebook — A detailed comparison of the different formulations of Active Inference found in the literature (2013-2019), including their mean-field and exact solutions in the general case of arbitrary many time steps. (HTML) [file pcbi.1008420.s005.html]

S1\_Notebook


# S1 Notebook: *Comparison of different formulations of Active Inference*¶

This notebook is published as supporting information accompanying the article *Gottwald, Braun. The two kinds of free energy and the Bayesian revolution*.

The structure of this notebook is as follows:

- Sections 1 and 2 compare recent formulations of *Active Inference* found in the literature (2013-2018),
- Section 3 presents mean-field solutions for each version in the general case of arbitrarily many time steps, and
- Section 4 presents their exact solutions.
- Section 5 lists some critical remarks

For each formulation listed below, changes from the preceding formulation are marked in red in order to quickly see the differences.

# 1. Commonalities among all versions of Active Inference listed below¶

---

#### *Assumed given distributions*¶

Likelihood $p\_0(X|S)$, prior over the initial state $p\_0(S\_0)$, transition probability $p\_0(S'|S,A)$, and a desired distribution $p\_\mathrm{des}$ which is either defined on future *states* (2013) or future *observations* (2015-2018).

#### *Random Variables*¶

The involved random variables are indexed by a time parameter $\tau\in \{0,\dots,T\}$,

$$
\mathbf{X} = (X\_\tau)\_{\tau=0}^T = (X\_0,\dots, X\_T) ,\quad \mathbf{S} = (S\_\tau)\_{\tau=0}^T =(S\_0,\dots,S\_{T}), \quad \mathbf{A} = (A\_\tau)\_{\tau=0}^{T-1} =(A\_0,\dots, A\_{T-1})
$$

We also use the shorthand notation $\mathbf{Y}\_t := (Y\_0,\dots,Y\_t)$, and more generally $\mathbf{Y}\_{t\_1:t\_2} := (Y\_{t\_1},\dots,Y\_{t\_2})$. In the case of paths over future variables exclusively, for readability we also write $\mathbf{Y}\_f := \mathbf{Y}\_{t+1:T}$ for future observations and states, and $\mathbf{A}\_f:=A\_{t:T-1}$ for future actions.

#### *Time dependency*¶

Even though this is not made explicit in the notation, all quantities (generative model, trial distributions, free energy, value function) depend on the current time $t$, i.e. they have to be re-determined for each new time step.

#### *Variational free energy*¶

The variational free energy serves as an error measure, which when minimized allows to fit trial distributions $q$ over unknown variables to a reference function $\phi$ that is constructed from the desired distribution $p\_\mathrm{des}$ and a generative model $p\_0$ (the full joint given by the fixed distributions),

$$
F = F(q\|\phi),
$$

In general, the reference function in a variational free energy expression does not have to be a normalized probability distribution, but in case it is then the variational free energy coincides with the relative entropy or Kullback-Leibler divergence $D\_\mathrm{KL}(q\|\phi)$. Here, the past observations $\mathbf{X}\_t = \mathbf{x}\_t$ and past actions $\mathbf{A}\_{t-1} = \mathbf{a}\_{t-1}$ must be fixed since they are no unknowns. The details of how $\phi$ is constructed from $p\_0$ and $p\_\mathrm{des}$ and thus the resulting free energy expressions differ between the versions listed below.

#### *Mean-field assumptions*¶

In most treatments of Active Inference, some kind of mean-field assumption is used to factorize the trial distributions $q$ over hidden variables in order to simplify the optimization procedure. In general, mean-field assumptions are strong simplifications that transform an optimization problem from a high-dimensional space to multiple coupled optimization problems over smaller dimensional spaces. Here, the coupling is created by an alternating optimization procedure, where free energy is optimized with respect to each factor separately while the other factors are kept fixed. This dependency is similar to the implicit dependency on other fixed quantities such as observations $\mathbf{x}$. In general, the coupling between the factors of mean-field solutions, say $q^\ast(A)$ and $q^\ast(S)$ is however much weaker than the coupling between the random variables $S$ and $A$ in an exact factorization of the form $q(A,S) = q(A)q(S|A)$, where $q(S|A)$ depends on the *values* of $A$ rather than on its *distribution* $q(A)$. In the case of very narrow distributions, i.e. $q(A)\approx \delta\_{A,a}$ for some value $a$, this differentiation becomes negligible (since the dependency on the value of $A$ and its distribution are then basically the same), which is why mean-field assumptions are successful in variational Bayesian inference if a single value of a certain hidden variable is able to explain the data well. However, in sequential decision-making problems, the coupling between the factors might be too weak, as can be seen in the simulations in Supporting Information *S2 Notebook*.

#### *Remark*¶

In all of the current Active Inference literature, the dependencies of the trial beliefs $q(S\_\tau|...)$ on actions is stated by simply writing $q(S\_\tau|\pi)$ where $\pi$ denotes the full action trajectory, or, equivalently, $\pi$ is a map $\tau\mapsto A\_\tau = \pi(\tau)$ from the time variable to actions. Instead of allowing the belief over states to depend on actions in the future, we have interpreted the notation $q(S\_\tau|\pi)$ as $q(S\_\tau|A\_t,\dots, A\_{\tau-1})$, so that beliefs over states can only depend on past actions (note that we omit the dependency of $q$ on already performed actions $a\_0,\dots,a\_{t-1}$ since they are treated like observations and are fixed during free energy optimization). In the grid world simulations the behavior of both variants is indistinguishable. We have also implemented an agent for the 2016/2017 version using the dependencies on the full trajectory, that can be tested in the grid world simulation in S2 Notebook (labelled `qmff`).

#### *Summary*¶

| Symbol | Meaning |
| --- | --- |
| $X$ | Observations |
| $S$ | Hidden states |
| $A$ | Actions |
| $p\_0$ | Generative model |
| $p\_\mathrm{des}$ | Desired distribution |
| $q$ | Trial distributions |
| $\phi$ | Reference function |

# 2. Differences¶

---

## **2013** - *Friston et al. The anatomy of choice, active inference and agency*¶

In this paper the desired distribution $p\_\mathrm{des}(S')$ is defined on future *states* $S'$, whereas in the rest of the versions listed below, the desired distribution is defined on future *observations* $X'$.

#### *Desired distribution*¶

$$
p\_\mathrm{des}(S')
$$

#### *Generative model (HMM)*¶

$$
p\_0(\mathbf{X}\_t,\mathbf{S}\_t|\mathbf{A}\_{t-1}) = \left(\prod\_{\tau = 0}^t p\_0(X\_\tau|S\_\tau) \right) \ p\_0(S\_0) \left (\prod\_{\tau=1}^t p\_0(S\_\tau|S\_{\tau-1},A\_{\tau-1})\right)
$$

#### *Reference*¶

The desired distribution $p\_\mathrm{des}$ and the generative model $p\_0$ are combined via a value function $Q$ to define the reference function

$$
\phi(\mathbf{X}\_t,\mathbf{S}\_t,\mathbf{A}) = p\_0(\mathbf{X}\_t,\mathbf{S}\_t|\mathbf{A}\_{t-1}) \, e^{Q(\mathbf{A}\_f,S\_t)}
$$

#### *Value function*¶

High value actions predict future states $S'$ that behave according to the desired distributions $p\_\mathrm{des}(S')$,

$$
Q(\mathbf{A}\_f,S\_t) = \left\langle \log \frac{p\_\mathrm{des}(S\_T)}{p\_0(S\_T|S\_t,\mathbf{A}\_f)} \right\rangle\_{p\_0(S\_T|S\_t,\mathbf{A}\_f)} = - D\_\mathrm{KL}\big(p\_0(S\_T|S\_t,\mathbf{A}\_f)\| p\_\mathrm{des}(S\_T) \big)
$$

where $p\_0(S\_T|S\_t,\mathbf{A}\_f) = \sum\_{s\_{t+1},...,s\_{T-1}} p\_0(S\_T|s\_{T-1},A\_{T-1})\cdots p\_0(s\_{t+1}|S\_{t},A\_{t})$.

#### *Trial distributions*¶

$$
q(\mathbf{S}\_t,\mathbf{A}\_f) = q(\mathbf{A}\_f) \prod\_{\tau = 0}^t q(S\_\tau) \, .
$$

#### *Variational free energy*¶

For a given set of observations $\mathbf{X}\_t = \mathbf{x}\_t$ and past actions $\mathbf{A}\_{t-1} = \mathbf{a}\_{t-1}$,

$$
F(q\|\phi) = \left\langle \log \frac{q(\mathbf{S}\_t,\mathbf{A}\_f)}{\phi(\mathbf{x}\_t,\mathbf{S}\_t,(\mathbf{a}\_{t-1},\mathbf{A}\_f))} \right\rangle\_{q(\mathbf{S}\_t,\mathbf{A}\_f)} \\
\qquad \qquad = \underbrace{\left\langle \log \frac{q(\mathbf{S}\_t)}{p\_0(\mathbf{x}\_t,\mathbf{S}\_t|\mathbf{a}\_{t-1})} \right\rangle\_{q(\mathbf{S}\_t)}}\_{=:\mathcal F\_{\mathbf{S}\_t}(\mathbf{x}\_t,\mathbf{a}\_{t-1})}
+ \left\langle D\_\mathrm{KL}\big(q(\mathbf{A}\_f)\| e^{Q(\mathbf{A}\_f,S\_t)}\big) \right\rangle\_{q(S\_t)}
$$

where $\mathcal F\_{\mathbf{S}\_t}(\mathbf{x}\_t,\mathbf{a}\_{t-1}) := F(q(\mathbf{S}\_t)\|p\_0(\mathbf{x}\_t,\mathbf{S}\_t|\mathbf{a}\_{t-1}))$ denotes the variational free energy over states only.

---

## **2015** - *Friston et al. Active inference and epistemic value*¶

This paper uses a desired distribution $p\_\mathrm{des}(X')$ over future *observations* $X'$ for the first time. It modifies the 2013 version in the definition of the value function $Q$ in two ways, it changes it to a sum over timesteps and more importantly, it incorporates the desired distribution in a way that uses the trial distributions $q(S\_t)$ over hidden states, which is conceptually problematic since minimizing the variational free energy over $q$ then also changes the reference distribution.

#### *Desired distribution*¶

Has changed to be defined over observations instead of states,

$$
p\_\mathrm{des}(\color{red}{X'})
$$

#### *Generative model, trial distributions*¶

Same as in the 2013 version.

#### *Reference*¶

Has the same shape as in the 2013 version, but with a value function $Q$ that is no longer a function of the current state,

$$
\phi(\mathbf{X}\_t,\mathbf{S}\_t,\mathbf{A}) = p\_0(\mathbf{X}\_t,\mathbf{S}\_t|\mathbf{A}\_{t-1}) \, e^{\color{red}{Q(\mathbf{A}\_f)}}
$$

#### *Value function*¶

The value function is now defined by

$$
\color{red}{Q(\mathbf{A}\_f)} = \color{red}{\sum\_{\tau=t+1}^T Q\_\tau(\mathbf{A}\_{t:\tau-1})}, \\
Q\_\tau(\mathbf{A}\_{t:\tau-1}) = \left\langle \log \frac{\color{red}{p\_0(X\_\tau|S\_\tau)} \, p\_\mathrm{des}(\color{red}{X\_\tau})}{\color{red}{\sum\_{s\_\tau} p\_0(X\_\tau|s\_\tau) \sum\_{s\_t}}p\_0(s\_\tau|s\_t,\mathbf{A}\_{t:\tau-1})\, \color{red}{q(s\_t)}}\right\rangle\_{\color{red}{p\_0(X\_\tau|S\_\tau) \sum\_{s\_t}}p\_0(S\_\tau|s\_t,\mathbf{A}\_{t:\tau-1}) \, \color{red}{q(s\_t)}}
$$

where $p\_0(S\_\tau|S\_t,\mathbf{A}\_{t:\tau-1}) = \sum\_{s\_{t+1},...,s\_{\tau-1}} p\_0(S\_\tau|s\_{\tau-1},A\_{\tau-1})\cdots p\_0(s\_{t+1}|S\_{t},A\_{t})$.

#### *Variational free energy*¶

For a given set of observations $\mathbf{X}\_t = \mathbf{x}\_t$ and past actions $\mathbf{A}\_{t-1} = \mathbf{a}\_{t-1}$,

$$
F(q\|\phi) = \left\langle \log \frac{q(\mathbf{S}\_t,\mathbf{A}\_f)}{\phi(\mathbf{x}\_t,\mathbf{S},(\mathbf{a}\_{t-1},\mathbf{A}\_f))} \right\rangle\_{q(\mathbf{S}\_t,\mathbf{A}\_f)} = \mathcal F\_{\mathbf{S}\_t}(\mathbf{x}\_t,\mathbf{a}\_{t-1})
+ D\_\mathrm{KL}\big(q(\mathbf{A}\_f)\| e^{\color{red}{Q(\mathbf{A}\_f)}}\big)
$$

where, as above, $\mathcal F\_{\mathbf{S}\_t}(\mathbf{x}\_t,\mathbf{a}\_{t-1}) := F(q(\mathbf{S}\_t)\|p\_0(\mathbf{x}\_t,\mathbf{S}\_t|\mathbf{a}\_{t-1}))$ denotes a free energy over states only. Note that $Q$ also depends on $\color{red}{q(S\_t)}$, which is ignored in the derivations of the update equations in the Active Inference literature.

---

## 2016 - *Friston et al. Active Inference and Learning*, and 2017 - *Friston et al. Active Inference: A Process Theory*

Even though the focus of this paper is to allow quantities that are usually given and fixed (such as likelihoods and priors) to be learned, it also uses a version of Active Inference that has been modified from the 2015 version. Especially, the generative model now depends on the full state variable $\mathbf{S}$ containing past, current states but also *future* states. Moreover, the trial distributions over states are now allowed to depend on actions and replace the predictive distributions $p(S\_\tau|\mathbf{A}) = \sum\_{s\_t}p\_0(S\_\tau|s\_t,\mathbf{A}\_{t:\tau-1}) q(s\_t)$ that appeared in the value function $Q$ in the 2015 version. Under the new (weaker) mean-field assumption the state variables at different time steps are still assumed to be independent from each other.

#### *Desired distribution*¶

Same as in the 2015 version.

#### *Generative model*¶

Now depends on actions and states at *all* time steps,

$$
p\_0(\mathbf{X}\_t,\color{red}{\mathbf{S}}|\color{red}{\mathbf{A}}) = \left(\prod\_{\tau=0}^t p\_0(X\_\tau|S\_\tau)\right)\ p\_0(S\_0) \left( \prod\_{\tau = 1}^\color{red}{T} p\_0(S\_\tau|S\_{\tau-1},A\_{\tau-1}) \right)
$$

#### *Reference*¶

In addition to the exponentiated value function, a fixed prior over future actions $p\_0(\mathbf{A}\_f)$ is multiplied to the reference,

$$
\phi(\mathbf{X}\_t,\color{red}{\mathbf{S}},\mathbf{A}) = p\_0(\mathbf{X}\_t,\color{red}{\mathbf{S}}|\color{red}{\mathbf{A}})\, p\_0(\mathbf{A}\_f) \, e^{Q(\mathbf{A}\_f)}
$$

#### *Value function*¶

The predictive distribution $\sum\_{s\_t} p\_0(S\_\tau|s\_t,\mathbf{A}\_{t:\tau-1}) q(s\_t)$ from the 2015 version is now replaced by the trial distribution $q(S\_\tau|\mathbf{A}\_{t:\tau-1})$,

$$
Q(\mathbf{A}\_f) = \sum\_{\tau = t+1}^T Q\_\tau(\mathbf{A}\_{t:\tau-1}), \quad
Q\_\tau(\mathbf{A}\_{t:\tau-1}) = \left\langle \log \frac{p\_0(X\_\tau|S\_\tau) p\_\mathrm{des}(X\_\tau)}{\sum\_{s\_\tau}p\_0(X\_\tau|s\_\tau) \, \color{red}{q(s\_\tau|\mathbf{A}\_{t:\tau-1})}}\right\rangle\_{p\_0(X\_\tau|S\_\tau) \color{red}{q(S\_\tau|\mathbf{A}\_{t:\tau-1})}}
$$

#### *Trial distributions*¶

The new trial distributions over future states are allowed to depend on actions,

$$
q(\color{red}{\mathbf{S}},\mathbf{A}\_f) = q(\mathbf{A}\_f) \left( \prod\_{\tau=0}^t q(S\_\tau)\right) \color{red}{\left( \prod\_{\tau=t+1}^T q(S\_\tau|\mathbf{A}\_{t:\tau-1})\right)}
$$

#### *Variational free energy*¶

For a given set of observations $\mathbf{X}\_t = \mathbf{x}\_t$ and past actions $\mathbf{A}\_{t-1} = \mathbf{a}\_{t-1}$,

$$
F(q\|\phi) = \left\langle \log \frac{q(\color{red}{\mathbf{S}},\mathbf{A}\_f)}{\phi(\mathbf{x}\_t,\color{red}{\mathbf{S}},(\mathbf{a}\_{t-1},\mathbf{A}\_f))} \right\rangle\_{q(\color{red}{\mathbf{S}},\mathbf{A}\_f)}\\
\qquad \qquad \qquad \quad = \underbrace{\left\langle \log \frac{q(\color{red}{\mathbf{S}|\mathbf{A}\_f})}{p\_0(\mathbf{x}\_t,\color{red}{\mathbf{S}}|\mathbf{a}\_{t-1}, \color{red}{\mathbf{A}\_f})} \right\rangle\_{q(\color{red}{\mathbf{S}|\mathbf{A}\_f})\color{red}{q(A\_f)}}}\_{ \color{red}{\langle} \mathcal F\_{\color{red}{\mathbf{S}}}(\mathbf{x}\_t,\mathbf{a}\_{t-1},\color{red}{\mathbf{A}\_f})\color{red}{\rangle\_{q(\mathbf{A}\_f)}} }
+ D\_\mathrm{KL}\big(q(\mathbf{A}\_f)\| p\_0(\mathbf{A}\_f) \, e^{Q(\mathbf{A}\_f)}\big)
$$

where

$$
\mathcal F\_{\color{red}{\mathbf{S}}}(\mathbf{x}\_t,\mathbf{a}\_{t-1},\color{red}{\mathbf{A}\_f}) := F(q(\color{red}{\mathbf{S}|\mathbf{A}\_f})\|p\_0(\mathbf{x}\_t,\color{red}{\mathbf{S}}|\mathbf{a}\_{t-1},\color{red}{\mathbf{A}\_f}))
$$

is a free energy over states only, for fixed $\mathbf{A}\_f$. Note that $Q$ also depends on $\color{red}{q(S\_\tau|\mathbf{A}\_{t:\tau-1})}$ for $\tau>t$, which is ignored in the derivations of the update equations in the Active Inference literature.

---

*Remark:* For simplicity we have neglected the precision parameter $\gamma$ that was present in earlier versions of Active Inference but later disappeared. Originally, it has been multiplied to the value function $Q$ and was treated as an additional unknown variable itself. Also, we did not highlight the fixed prior $p\_0(\mathbf{A}\_f)$ that has been introduced in the 2016 version because this is a minor change that could have easily been there from the beginning.

## 2018 - *Schwöbel et al. Active Inference, Belief Propagation, and the Bethe Approximation*¶

#### *Desired distribution, generative model*¶

Same as in the 2016/2017 version.

#### *Reference*¶

Here, the desired distribution $p\_\mathrm{des}$ and the generative model $p\_0$ are combined by simply multiplying them without renormalization, which is a special case of *Control as Inference* (Toussaint 2006), where the extra factor is interpreted as the probability of an auxiliary success variable (see Section 5.3 $(iii)$ and S2 Notebook):

$$
\phi(\color{red}{\mathbf{X}},\mathbf{S},\mathbf{A}) = \color{red}{\left(\prod\_{\tau = t+1}^T p\_\mathrm{des}(X\_\tau) p\_0(X\_\tau|S\_\tau) \right)} p\_0(\mathbf{X}\_t,\mathbf{S}|\mathbf{A})\, p\_0(\mathbf{A}\_f)
$$

#### *Trial distributions* (in case of a partial mean-field assumption)¶

$$
q(\color{red}{\mathbf{X}\_f},\mathbf{S},\mathbf{A}\_f) = q(\mathbf{A}\_f) \left( \prod\_{\tau=0}^t q(S\_\tau)\right) \left( \prod\_{\tau=t+1}^T \color{red}{p\_0(X\_\tau|S\_\tau)}\, q(S\_\tau|\mathbf{A}\_{t:\tau-1})\right)
$$

#### *Variational free energy*¶

For a given set of observations $\mathbf{X}\_t = \mathbf{x}\_t$ and past actions $\mathbf{A}\_{t-1} = \mathbf{a}\_{t-1}$,

$$
F(q\|\phi) = \left\langle \log \frac{q(\color{red}{\mathbf{X}\_f},\mathbf{S},\mathbf{A}\_f)}{\phi((\mathbf{x}\_t,\color{red}{\mathbf{X}\_f}),\mathbf{S},(\mathbf{a}\_{t-1},\mathbf{A}\_f)) } \right\rangle\_{q(\color{red}{\mathbf{X}\_f},\mathbf{S},\mathbf{A}\_f)} \\
\qquad =
\color{red}{\big\langle -\log p\_\mathrm{des}(\mathbf{X}\_f) \big\rangle\_{p\_0(\mathbf{X}\_f|\mathbf{S}\_f)q(\mathbf{S}\_f|\mathbf{A}\_f)\,q(\mathbf{A}\_f)}}
+ \left\langle \log \frac{ q(\mathbf{S}|\mathbf{A}\_f)}{p\_0(\mathbf{x}\_t,\mathbf{S}|\mathbf{a}\_{t-1}, \mathbf{A}\_f)} \right\rangle\_{q(\mathbf{S}|\mathbf{A}\_f)q(A\_f)}
+ D\_\mathrm{KL}\big(q(\mathbf{A}\_f)\|p\_0(\mathbf{A}\_f)\big)
$$

---

# 3. Iterative Mean-Field Solutions¶

---

Here, we show the resulting mean-field solutions **when neglecting the extra dependency of $Q$ on trial distributions $q$** (in the 2015 and 2016/2017 versions), which is an assumption that is made in the Active Inference literature, but results in **very different behavior than when it is correctly included** (see S2 Notebook: Grid world simulations). The proportionality symbol $\propto$ means that the expressions are equal up to multiplicative constants, in particular, the right side has to be normalized with respect to the corresponding variable in order to be a probability distribution. Also, to simplify notation, the transition probabilities $p\_0(S\_\tau|S\_{\tau-1},A\_{\tau-1})$ also include the prior $p\_0(S\_0)$ for $\tau = 0$.

## **2013**¶

**States:**

For $\tau < t$,
$$
q^\ast(S\_\tau) \propto p\_0(x\_\tau|S\_\tau) \, \exp\left[\langle \log p\_0(S\_\tau|S\_{\tau-1},a\_{\tau-1})\rangle\_{q(S\_{\tau-1})} + \langle \log p\_0(S\_{\tau+1}|S\_\tau,a\_\tau)\rangle\_{q(S\_{\tau+1})}\right]
$$

whereas for $\tau = t$,

$$
q^\ast(S\_t) \propto p\_0(x\_t|S\_t) \, \exp\left[\langle \log p\_0(S\_t|S\_{t-1},a\_{t-1})\rangle\_{q(S\_{t-1})} + \langle Q(\mathbf{A}\_f,S\_t)\rangle\_{q(\mathbf{A}\_f)} \right]
$$

**Actions:**

$$
q(\mathbf{A}\_f) \propto e^{\langle Q(\mathbf{A}\_f,S\_t)\rangle\_{q(S\_t)}}
$$

where $Q(\mathbf{A}\_f,S\_t) = - D\_\mathrm{KL}(p\_0(S\_T|S\_t,\mathbf{A}\_{t:T-1})\|p\_\mathrm{des}(S\_T))$ and $p\_0(S\_T|S\_t,\mathbf{A}\_{t:T-1}) = \sum\_{s\_{T-1}} \cdots \sum\_{s\_{t+1}} p\_0(S\_T|s\_{T-1}, A\_{T-1}) \cdots p\_0(s\_{t+1}|S\_t,A\_t)$.

## **2015**¶

**States:**

For $\tau < t$, $q^\ast(S\_\tau)$ has the same shape as in the 2013 version, whereas for $\tau = t$,

$$
q^\ast(S\_t) \propto p\_0(x\_t|S\_t) \color{red}{\exp\left[\langle \log p\_0(S\_t|S\_{t-1},a\_{t-1})\rangle\_{q(S\_{t-1})}\right]}
$$

**Actions:**

$$
q^\ast(\mathbf{A}\_f) \propto e^{\color{red}{Q(\mathbf{A}\_f)}}
$$

where

$$
\color{red}{
Q(\mathbf{A}\_f) = \sum\_{\tau=t+1}^T \mathcal Q[p(S\_\tau|\mathbf{A}\_{t:\tau-1})], \quad \mathcal Q[q(S)] := \left\langle \log \frac{p\_0(X|S) p\_\mathrm{des}(X)}{\sum\_{s} p\_0(X|s) q(s)} \right\rangle\_{p\_0(X|S)q(S)}}$$

with the predictive distribution

$$
\color{red}{p(S\_\tau|\mathbf{A}\_{t:\tau-1})} = \color{red}{\sum\_{s\_t}} p\_0(S\_\tau|s\_t,\mathbf{A}\_{t:\tau-1})\, \color{red}{q(s\_t)} = \color{red}{\sum\_{s\_t}} \sum\_{s\_{\tau-1}} \cdots \sum\_{s\_{t+1}} p\_0(S\_\tau|s\_{\tau-1}, A\_{\tau-1}) \cdots p\_0(s\_{t+1}|S\_t,A\_t) \, \color{red}{q(s\_t)}
$$

## **2016/2017**¶

**States:**
For $\tau< t$, $q^\ast(S\_\tau)$ has the same shape as in the 2013 and 2015 versions, whereas for $\tau = t$,

$$
q^\ast(S\_t) \propto p\_0(x\_t|S\_t) \exp\left[\langle \log p\_0(S\_t|S\_{t-1},a\_{t-1})\rangle\_{q(S\_{t-1})} + \color{red}{\langle \log p\_0(S\_{t+1}|S\_t,A\_t)\rangle\_{q(S\_{t+1}|A\_t)\,q(A\_t)}} \right]
$$

and for $\tau>t$,
$$\color{red}{q^\ast(S\_\tau|\mathbf{A}\_{t:\tau-1})
\propto \exp\left[\langle \log p\_0(S\_\tau|S\_{\tau-1},A\_{\tau-1})\rangle\_{q(S\_{\tau-1}|\mathbf{A}\_{t:\tau-2})} +\, \langle \log p\_0(S\_{\tau+1}|S\_{\tau},A\_{\tau})\rangle\_{q(S\_{\tau+1}|A\_{t:\tau})q(A\_\tau)}\right]}
$$

**Actions:**
$$
q^\ast(\mathbf{A}\_f) \propto p\_0(\mathbf{A}\_f) \, e^{\color{red}{-\mathcal F\_{\mathbf{S}}(\mathbf{x}\_t,\mathbf{a}\_{t-1},\mathbf{A}\_f)} + Q(\mathbf{A}\_f)}
$$

where

$$
\color{red}{
\mathcal F\_{\mathbf{S}}(\mathbf{x}\_t,\mathbf{a}\_{t-1},\mathbf{A}\_f) = \sum\_{\tau=t+1}^{T} \left\langle \log \frac{q(S\_{\tau}|\mathbf{A}\_{t:\tau-1})}{p\_0(S\_\tau|S\_{\tau-1},A\_{\tau-1})} \right\rangle\_{q(S\_\tau|\mathbf{A}\_{t:\tau-1})q(S\_{\tau-1}|\mathbf{A}\_{t:\tau-2})} + \underbrace{f(\mathbf{x}\_t,\mathbf{a}\_{t-1})}\_{\text{constant}}}
$$

and

$$
Q(\mathbf{A}\_f) = \sum\_{\tau=t+1}^T \mathcal Q[\color{red}{q(S\_\tau|\mathbf{A}\_{t:\tau-1})}],
$$

with $\mathcal Q[q(S)]$ as defined in the 2015 version.

## 2018¶

**States:**
For $\tau\leq t$, $q^\ast(S\_\tau)$ has the same shape as the 2016/2017 version, whereas for $\tau>t$,

$$q^\ast(S\_\tau|\mathbf{A}\_{t:\tau-1})
\propto \exp\Big[\langle \log p\_0(S\_\tau|S\_{\tau-1},A\_{\tau-1})\rangle\_{q(S\_{\tau-1}|\mathbf{A}\_{t:\tau-2})} +\, \langle \log p\_0(S\_{\tau+1}|S\_{\tau},A\_{\tau})\rangle\_{q(S\_{\tau+1}|A\_{t:\tau})q(A\_\tau)}+\color{red}{\langle \log p\_\mathrm{des}(X\_\tau)\rangle\_{p\_0(X\_\tau|S\_\tau)}} \Big]
$$

**Actions:**

$$
q^\ast(\mathbf{A}\_f) \propto p\_0(\mathbf{A}\_f) \, e^{-\mathcal F\_{\mathbf{S}}(\mathbf{x}\_t,\mathbf{a}\_{t-1},\mathbf{A}\_f) \color{red}{+ D(\mathbf{A}\_f)}}
$$

where

$$
\color{red}{D(\mathbf{A}\_f) := \sum\_{\tau=t+1}^T \left\langle \log p\_\mathrm{des}(X\_\tau) \right \rangle\_{p\_0(X\_\tau|S\_\tau)q(S\_\tau|\mathbf{A}\_{t:\tau-1})}}
$$

# 4. Exact Solutions¶

---

Here, we show the trial distributions that would minimize the various free energies perfectly. **The variational solutions shown above are supposed to approximate these exact solutions**.

In general, as follows directly from Jensen's inequality, the exact solution to a variational free energy problem of the form
$$
\min\_q F(q\|\phi) = \min\_q \left \langle \log \frac{q(Z)}{\phi(Z)} \right \rangle\_{q(Z)}
$$
is given by
$$
q^\ast(Z) = \frac{\phi(Z)}{\sum\_z \phi(z)} ,
$$

i.e. it is simply the normalized version of the reference $\phi$. One issue of the 2015 and 2016 versions of Active Inference is that the value function $Q$ that is part of the reference $\phi$ depends on trial distributions $q$, which means that the reference $\phi$ is not constant anymore when $q$ is varied (see the discussion in $(ii)$ of Section 5.3 of the article). In this section, we handle this problem by using the exact solution $q^\ast$ instead of the mean-field solutions to determine the value function $Q$, which is possible since the optimization problem over actions and states splits into two separate problems anyways.

The goal of Active Inference is to choose actions that lead to a desired future specified by $p\_\mathrm{des}$. Therefore, in the following, it is sufficient to calculate the exact posteriors over actions, whereas the state distributions are only inferred if they are required to determine these action posteriors.

## **2013**¶

The exact solutions in the original version of Active Inference cannot be simplified much, due to the extra dependency on $S\_t$ in the value function:

$$
q^\ast(\mathbf{S}\_t,\mathbf{A}\_f) = \frac{\phi(\mathbf{x}\_t,\mathbf{S}\_t,(\mathbf{a}\_{t-1},\mathbf{A}\_f))}{\sum\_{\mathbf{s}\_t,\mathbf{a}\_f}\phi(\mathbf{x}\_t,\mathbf{s}\_t,(\mathbf{a}\_{t-1},\mathbf{a}\_f))} = \frac{e^{Q(\mathbf{A}\_f,S\_t)} p\_0(\mathbf{x}\_t,\mathbf{S}\_t|\mathbf{a}\_{t-1})}{ \sum\_{\mathbf{a}\_f}\sum\_{s\_t} e^{Q(\mathbf{a}\_f,s\_t)} \sum\_{\mathbf{s}\_{t-1}} p\_0(\mathbf{x}\_t,\mathbf{s}\_t|\mathbf{a}\_{t-1}) }
$$

The action distribution is now given by the marginal,
$$
q^\ast(\mathbf{A}\_f) = \sum\_{\mathbf{s}\_t} q^\ast(\mathbf{s}\_t,\mathbf{A}\_f)
$$

## **2015**¶

$$
q^\ast(\mathbf{S}\_t,\mathbf{A}\_f) = \frac{\phi(\mathbf{x}\_t,\mathbf{S}\_t,(\mathbf{a}\_{t-1},\mathbf{A}\_f))}{\sum\_{\mathbf{s}\_t,\mathbf{a}\_f}\phi(\mathbf{x}\_t,\mathbf{s}\_t,(\mathbf{a}\_{t-1},\mathbf{a}\_f))} = \frac{e^{\color{red}{Q(\mathbf{A}\_f)}}}{\sum\_{\mathbf{a}\_f}e^{\color{red}{Q(\mathbf{a}\_f)}}} \frac{p\_0(\mathbf{x}\_t,\mathbf{S}\_t|\mathbf{a}\_{t-1})}{\color{red}{\sum\_{\mathbf{s}\_{t}}} p\_0(\mathbf{x}\_t,\mathbf{s}\_t|\mathbf{a}\_{t-1}) }
$$

Due to the separation into states $\mathbf{S}\_t$ and future actions $\mathbf{A}\_f$, the action marginal distribution is simply given by the first factor

$$
q^\ast(\mathbf{A}\_f) = \sum\_{\mathbf{s}\_t} q^\ast(\mathbf{s}\_t,\mathbf{A}\_f) \color{red}{= \frac{e^{Q(\mathbf{A}\_f)}}{\sum\_{\mathbf{a}\_f}e^{Q(\mathbf{a}\_f)}}}
$$

where the calculation of $Q$ requires the distribution over the current state $q^\ast(S\_t)$ which can be obtained from

$$
q^\ast(\mathbf{S}\_t) = \sum\_{\mathbf{a}\_f} q^\ast(\mathbf{S}\_t,\mathbf{a}\_f) \color{red}{ =\frac{p\_0(\mathbf{x}\_t,\mathbf{S}\_t|\mathbf{a}\_{t-1})}{ \sum\_{\mathbf{s}\_{t}} p\_0(\mathbf{x}\_t,\mathbf{s}\_t|\mathbf{a}\_{t-1}) }}
$$

which is Bayes rule defining the Bayes posterior $p\_0(\mathbf{S}\_t|\mathbf{x}\_t,\mathbf{a}\_{t-1})$.
Hence, the Active Inference problem in this version can be solved *exactly* by doing Bayesian inference over the states $\mathbf{S}\_t$ allowing to calculate the marginal $q^\ast(S\_t) = p\_0(S\_t|\mathbf{x}\_t,\mathbf{a}\_{t-1})$ which is required to evaluate the value function $Q(\mathbf{A}\_f)$. In particular, the exact solution of Active Inference in this case consists in the pre-specified action distribution defined by the softmax function of $Q(\mathbf{A}\_f)$, which is determined using Bayesian inference.

## **2016/2017**¶

The exact solution to this version is equivalent to the 2015 version, since the exact posteriors $q^\ast(S\_\tau|\mathbf{A}\_{t:\tau-1})$ here are given by the predictive versions $p\_0(S\_\tau|\mathbf{x}\_t,\mathbf{a}\_{t-1},\mathbf{A}\_{t:T-1})$ of the Bayes posteriors $p\_0(S\_t|\mathbf{x}\_t,\mathbf{a}\_{t-1})$ using the transition probabilities $p\_0(S'|S,A)$.

## 2018¶

The exact solution to the 2018 version is **a special case of *Control as Inference*** (e.g. Toussaint 2006) that is well-known in the machine learning literature, see S2 Notebook for an implementation in a grid world simulation.

# 5. Critical Remarks (see Section 5.3 of the article for more details)¶

---

- **Combining $p\_\mathrm{des}$ and $p\_0$ is not unique:** In all $Q$-value versions of Active Inference (2013-2017), the desired distribution is put into the reference via a specifically designed value function, whereas in direct Active Inference (2018), it is directly multiplied to the reference model. In general, there is no unique approach of how to combine the desired distribution with the generative model.

- **The reference depends on $q$:** The reference functions in most Active Inference approaches using a value function $Q$ depend (non-linearly) on trial distributions $q$, $\phi=\phi(q)$, in particular they also change during free energy optimization with respect to $q$. This is both, conceptually and practically problematic:

  - In variational Bayesian inference, free energy minimization corresponds to trial distributions being fitted to a *constant* reference $\phi$ (given by the probabilistic model evaluated under the quantities to be conditioned on). In Active Inference, the reference itself changes during free energy minimization, which means that it is not clear anymore what is actually approximated by the trial distributions.
  - In practice, this extra dependency on $q$ is ignored by the Active Inference update equations. However, as can be seen in the simulations in S2 Notebook, when this dependency is included then the resulting behavior is very different and not goal-directed at all, reflecting the problem that the reference itself changes during the optimization which leads to unpredictable solutions.

- **Conditioning on the past can only reproduce presupposed distributions:** In $Q$-value Active Inference (2013-2017), one essentially conditions on past experience, which can only reproduce action distributions that are put into the reference. This means that the form of *the action distribution is predefined*.

In [ ]:

```

```
