# Supplementary material for: The two kinds of free energy and the Bayesian revolution
Source: PLoS Comput Biol. 2020 Dec 3;16(12):e1008420. doi: 10.1371/journal.pcbi.1008420 (PMC7714236; doi:10.1371/journal.pcbi.1008420)
Supplement: S2 Notebook — We provide implementations of the models discussed in this article in a grid world environment, both as a rendered html file as well as a jupyter notebook that is available on github. (HTML) [file pcbi.1008420.s006.html]

S2\_Notebook


In [1]:

```
# imports and config↔
```

In [2]:

```
%%html↔
```

# S2 Notebook: Grid world simulations¶

This notebook is published as supporting information accompanying the article *Gottwald, Braun. The two kinds of free energy and the Bayesian revolution*. This is a rendered version of the jupyter notebook available on github using animated gif images instead of real simulations.

# 1. *Simulations*¶

Below we have implemented various inference-based agents discussed in the article in a simple grid world environment. The grid world consists of a square with two inner wall blocks and two targets. One of the targets is covered in a region of bad visibility (almost uniform $p\_0(X|S)$), visualized with a gray background. The transition probability is assumed to be perfect (i.e. $p\_0(S'|S,A)$ is a Dirac distribution given by the grid). Note that the grid world setup can be changed freely in the jupyter notebook on github.

In order to be able to compare the pure inference-based and utility-based approaches with Active Inference, we have assumed a desired distribution for all methods. However, as discussed in the article, the approaches differ in how they treat such a distribution, e.g. $p\_\mathrm{des}(X)$ might be viewed as a success probability $p(R=1|X)$ (when conditioning on success), or $\log p\_\mathrm{des}(X)$ as a utility $U(X)$. In the simulations below where a soft-maximization appears, we have set the optional precision parameter $\alpha=1$. One can easily test other values in the available juypter notebook, but for this simple environment higher values seem to make little to no difference (lower values result in more random behavior, of course).

In [3]:

```
# define environment (jupyter notebook only)↔
```

## Overview: *Approaches using exact inference*¶

For the following overview of the resulting behavior of the different agents, we have performed 1000 trials. We display the eight most probable paths for each agent (or all in case there are less than 8 different ones), where we use differently dotted lines for each path, with the density of dots indicating the rank in the probability rating.

In [4]:

```
# load trajectories from file and show most probable ones↔
```

## Overview: *Approaches using variational/approximate inference*¶

In [5]:

```
# load trajectories from file and show most probable ones↔
```

---

In the following we write $\mathrm{Tr}\_{s' s}^a$ as a shorthand for the transition probability $p\_0(s'|s,a)$, and "$\text{norm.}$" to indicate normalization (a function of the variables in the conditional of the resulting distribution only).

## I. Conditioning on $p\_\mathrm{des}$ (Jeffrey conditionalization)¶

The following approach first samples a desired state $x\_\mathrm{des}$ from the desired distribution $p\_\mathrm{des}$ and then uses exact Bayesian inference over actions conditioned on $X\_T = x\_\mathrm{des}$.

#### for $t=0,\dots,T-1$:¶

(1) Observe $X\_t=x\_t$ and sample a desired observation $x\_\mathrm{des} \sim p\_\mathrm{des}(X)$

(2) Inference over states:

$\qquad p(S\_t|\mathbf{x}\_t,\mathbf{a}\_{t-1}) \leftarrow \left\{ \begin{array}{cc} \frac{1}{\text{norm.}}\, p\_0(x\_0|S\_0) p\_0(S\_0) & \text{if } t=0 \\ \frac{1}{\text{norm.}} \, p\_0(x\_t|S\_t) p(S\_t|\mathbf{x}\_{t-1},\mathbf{a}\_{t-1}) & \text{if } t>0 \end{array}\right. $

where $p(S\_t|\mathbf{x}\_{t-1},\mathbf{a}\_{t-1}) = \sum\_{s\_{t-1}} \mathrm{Tr}\_{S\_t s\_{t-1}}^{a\_{t-1}} p(s\_{t-1}|\mathbf{x}\_{t-1},\mathbf{a}\_{t-2})$ is determined using the Bayes posterior $p(S\_{t-1}|\mathbf{x}\_{t-1},\mathbf{a}\_{t-2})$ from the previous timestep.

(3) Inference over actions:

$$
p(A\_t,\dots,A\_{T-1}|X\_T = x\_\mathrm{des},\mathbf{x}\_t,\mathbf{a}\_{t-1}) = \frac{1}{\text{norm.}} \, \sum\_{s\_T}p\_0(x\_\mathrm{des}|s\_{T}) \sum\_{s\_t,\dots,s\_{T-1}} \mathrm{Tr}\_{s\_T s\_{T-1}}^{A\_{T-1}} \cdots \mathrm{Tr}\_{s\_{t+1} s\_t}^{A\_t} \, p(s\_t|\mathbf{x}\_t,\mathbf{a}\_{t-1})
$$

(4) Sample $a\_t$ from $\sum\_{a\_{t+1},\dots,a\_{T-1}} p(A\_t,a\_{t+1},\dots,a\_{T-1}|x\_\mathrm{des})$ or sample a full trajectory and ignore everything but $a\_t$.

Note that this corresponds to Jeffrey conditioning as it produces actions sampled from $\sum\_{x\_T} p(\mathbf{A}\_f|x\_T) p\_\mathrm{des}(x\_T)$.

In [5]:

```
# show sample behavior↔
```

Out[5]:

In [ ]:

```
# set up grid world (jupyter notebook only)↔
```

In [8]:

```
# run the agent (jupyter notebook only)↔
```

```
Navigating...Done: Success after 5 steps!
Navigating...Fail: Episode stopped after 5 steps
Navigating...Fail: Episode stopped after 5 steps
```

---

## II. Conditioning on success (Control as inference, e.g. Toussaint 2006)¶

The other option to perform inference over actions is to introduce an auxiliary variable $R$ which encodes the occurence of desired outcomes $x\_\mathrm{des}$ by assuming a probability $p(R=1|X\_\tau=x\_\mathrm{des})$, usually defined through a given reward function (cf. Section 5.3 $(iii)$ of the article).

#### for $t=0,\dots,T-1$:¶

(1) Observe $X\_t=x\_t$.

(2) Inference over states: $p(S\_t|\mathbf{x}\_t,\mathbf{a}\_{t-1})$ same as in I.

(3) Inference over actions: Introduce a binary success variable $R$ so that $R=1$ encodes the occurence of desired outcomes, for example by using a conditional of the form $p\_0(R=1|X\_T=x\_T) := p\_\mathrm{des}(x\_T)$, which is multiplied to the full joint $p\_0$ to obtain a new joint $p\_0(R,\mathbf{X}\_{t+1:T},\mathbf{S},\mathbf{A}\_{t:T-1}|\mathbf{x}\_t,\mathbf{a}\_{t-1})$. Success can now be considered an observable on which can be conditioned to obtain a distribution over inferred action trajectories:

$$
p(A\_t,\dots,A\_{T-1}|R=1,\mathbf{x}\_t,\mathbf{a}\_{t-1}) = \frac{1}{\text{norm.}} \, \sum\_{x\_T} p\_0(R=1|x\_T) \sum\_{s\_T} p\_0(x\_T|s\_T)\,\sum\_{s\_t,\dots,s\_{T-1}} \mathrm{Tr}\_{s\_T s\_{T-1}}^{A\_{T-1}} \cdots \mathrm{Tr}\_{s\_{t+1} s\_t}^{A\_t} \, p(s\_t|\mathbf{x}\_t,\mathbf{a}\_{t-1})
$$

(4) Sample $a\_t$ from $\sum\_{a\_{t+1},\dots,a\_{T-1}} p(A\_t,a\_{t+1},\dots,a\_{T-1}|R=1,\mathbf{x}\_t,\mathbf{a}\_{t-1})$ or sample a full trajectory and ignore everything but $a\_t$.

Remark: One can also get rid of the $T$-dependency by treating $T$ as a random variable with a distribution of the form $p\_0(T) \propto \gamma^T$ for some $0<\gamma<1$.

In [6]:

```
# show sample behavior↔
```

Out[6]:

In [ ]:

```
# set up grid world (jupyter notebook only)↔
```

In [13]:

```
# run the agent (jupyter notebook only)↔
```

```
Navigating...Done: Success after 5 steps!
Navigating...Done: Success after 5 steps!
Navigating...Done: Success after 5 steps!
```

---

## III. Soft-maximizing expected utility¶

#### for $t=0,\dots,T-1$:¶

(1) Observe $X\_t=x\_t$.

(2) Same as in I.

(3) Calculate soft-max action policy (with precision parameter $\alpha$):

$$
p(\mathbf{A}\_{t:T-1}) \leftarrow \frac{1}{\text{norm.}}\, p\_0(\mathbf{A}\_{0:T-1}) \, e^{\alpha\sum\_{\tau=t+1}^T \langle U(X\_\tau)\rangle\_{p\_0(X\_\tau|S\_\tau)p(S\_\tau|\mathbf{A}\_{t:\tau-1})}}
$$

where $U$ is a given utility function over future observations (e.g. $U(x)=\delta\_{x,x\_\mathrm{des}}$ in the gridworld example below)

$$
p(S\_\tau|\mathbf{A}\_{t:\tau-1}) = \sum\_{s\_t,\dots,s\_{\tau-1}} \mathrm{Tr}\_{S\_\tau s\_{\tau-1}}^{A\_{\tau-1}} \cdots \mathrm{Tr}\_{s\_{t+1} s\_t}^{A\_t} p(s\_t|\mathbf{x}\_t,\mathbf{a}\_{t-1})
$$

(4) Sample $a\_t$ from $\sum\_{a\_{t+1},\dots,a\_{T-1}} p(A\_t,a\_{t+1},\dots,a\_{T-1})$ or sample a full trajectory and ignore everything but $a\_t$.

Note that, here we have implemented a very simple version of a utility-based agent with information constraints, where the precision-parameter $\alpha$ in the soft-maximization (3.) controls the trade-off between maximizing expected utility and staying close to the prior $p\_0(A)$ (a constraint on the KL divergence between $q(A)$ and $p\_0(A)$). In this simple environment, a choice of $\alpha=1$ (which we have chosen for all the simulations with a soft-max in this notebook) is pretty much equivalent to a rational agent that maximizes expected utility without any constraints ($\alpha\to\infty$). See e.g. Grau-Moya et al. 2016 for grid world simulations that specifically target robust behaviour under model uncertainty and information constraints.

In [7]:

```
# show sample behavior↔
```

Out[7]:

In [ ]:

```
# set up grid world (jupyter notebook only)↔
```

In [16]:

```
# run the agent (jupyter notebook only)↔
```

```
Navigating...Done: Success after 5 steps!
Navigating...Done: Success after 5 steps!
Navigating...Done: Success after 5 steps!
```

---

## IV. Q-value Active Inference (Friston et al. 2016/2017)¶

Here, we have implemented the 2016/2017 version of Active Inference based on the partial mean-field assumption, which is the most capable one among the $Q$-value versions of Active Inference (2013-2017). However, as we can see in the simulation below, even the partial mean-field assumption is too restrictive to plan actions in a simple grid world. Moreover, following the literature, the $q$-dependency of $Q$ was ignored in the derivation of the update equations. In IV.b, we show that the resulting behavior differs when correctly considering the $q$-dependency $Q=Q(q)$ in the update steps (using gradient descent). In IV.c, we show the predefined "prior" behavior that a simple soft-max policy using the *exact* value function $Q=Q(p\_\mathrm{pred})$ would achieve (note that, in the case of our idealized grid world environment, the exact solution requires less computational effort than the mean-field versions).

### IV.a *Mean-field approximation,* $Q=Q(q)$, *$q$-dependency ignored in gradients*¶

#### for $t=0,...,T-1$:¶

(1) observe $X\_t = x\_t$

(2) initialize $q(S\_0),\dots,q(S\_t),q(S\_{t+1}|A\_t),\dots,q(S\_T|A\_t,\dots,A\_{T-1})$, and $q(A\_t,\dots,A\_{T-1})$.

(3) repeat until convergence:

$$
q(S\_0) \leftarrow \frac{1}{\text{norm.}} \, p\_0(S\_0) \, p\_0(x\_0|S\_0) \left\{ \begin{array}{cc} e^{\langle \log \mathrm{Tr}\_{S\_1,S\_0}^{A\_0}\rangle\_{q(S\_1|A\_0)q(A\_0)}} & \text{if } t=0 \\ e^{\langle \log \mathrm{Tr}\_{S\_1 S\_0}^{a\_0}\rangle\_{q(S\_1)}} & \text{if } t>0 \end{array}\right.
$$

for $\tau=1,\dots,T-1$:

$$
\qquad q(S\_\tau|\mathbf{A}\_{t:\tau-1}) \leftarrow \left\{ \begin{array}{cc}\frac{1}{\text{norm.}}\, p\_0(x\_\tau|S\_\tau) \, e^{\langle \log \mathrm{Tr}\_{S\_\tau S\_{\tau-1}}^{a\_{\tau-1}}\rangle\_{q(S\_{\tau-1})}} e^{ \langle \log \mathrm{Tr}\_{S\_{\tau+1}S\_{\tau}}^{a\_{\tau}}\rangle\_{q(S\_{\tau+1})}} & \text{if } \tau<t \\
\frac{1}{\text{norm.}} \, p\_0(x\_t|S\_t) e^{\langle \log \mathrm{Tr}\_{S\_t S\_{t-1}}^{a\_{t-1}} \rangle\_{q(S\_{t-1})}} e^{\langle \log \mathrm{Tr}\_{S\_{t+1} S\_t}^{A\_t}\rangle\_{q(S\_{t+1}|A\_t) q(A\_t)}} & \text{if } \tau = t \\
\frac{1}{\text{norm.}} \,e^{\langle \log \mathrm{Tr}\_{S\_\tau S\_{\tau-1}}^{A\_{\tau-1}}\rangle\_{q(S\_{\tau-1}|A\_t,\dots,A\_{\tau-2})}} e^{ \langle \log \mathrm{Tr}\_{S\_{\tau+1}S\_{\tau}}^{A\_{\tau}}\rangle\_{q(S\_{\tau+1}|A\_t,\dots,A\_{\tau})q(A\_{\tau})} } & \text{if } \tau>t\end{array}\right.
$$

end for

$$
q(S\_T|\mathbf{A}\_{t:T-1}) \leftarrow \frac{1}{\text{norm.}} \, e^{\langle \log \mathrm{Tr}\_{S\_T S\_{T-1}}^{A\_{T-1}}\rangle\_{q(S\_{T-1}|A\_t,\dots,A\_{T-2})}}
$$$$
q(A\_t,\dots,A\_{T-1}) \leftarrow \frac{1}{\text{norm.}}\, p\_0(\mathbf{A}\_{t:T-1}) \, e^{\alpha \sum\_{\tau={t+1}}^T G\_\tau(\mathbf{A}\_{t:\tau-1})}
$$

where $G\_\tau(\mathbf{A}\_{t:\tau-1}) = -\mathcal F\_{\mathbf{S}}(\mathbf{A}\_{t:\tau-1}) + Q\_\tau(\mathbf{A}\_{t:\tau-1})$ is determined by

$$
\langle \log \mathrm{Tr}\_{S\_\tau,S\_{\tau-1}}^{A\_{\tau-1}} \rangle\_{q(S\_\tau|\mathbf{A}\_{t:\tau-1}) q(S\_{\tau-1}|\mathbf{A}\_{t:\tau-2})} - \langle \log q(S\_\tau|\mathbf{A}\_{t:\tau-1})\rangle\_{q(S\_\tau|\mathbf{A}\_{t:\tau-1})} + \langle \log p\_0(X\_\tau|S\_\tau)\rangle\_{p\_0(X\_\tau|S\_\tau)q(S\_\tau|\mathbf{A}\_{t:\tau-1})}\\
+ \langle \log p\_\mathrm{des}(X\_\tau)\rangle\_{p\_0(X\_\tau|S\_\tau)q(S\_\tau|\mathbf{A}\_{t:\tau-1})} - \left\langle \log \sum\_{s\_\tau} p\_0(X\_\tau|s\_\tau) q(s\_\tau|\mathbf{A}\_{t:\tau-1})\right\rangle\_{p\_0(X\_\tau|S\_\tau) q(S\_\tau|\mathbf{A}\_{t:\tau-1})}
$$

(4) Sample $a\_t$ from $\sum\_{a\_{t+1},\dots,a\_{T-1}} q(A\_t,a\_{t+1},\dots,a\_{T-1})$.

In [8]:

```
# show sample behavior↔
```

Out[8]:

In [ ]:

```
# set up grid world (jupyter notebook only)↔
```

In [12]:

```
# run the agent (jupyter notebook only)↔
```

```
Navigating...Fail: Episode stopped after 5 steps
Navigating...Fail: Episode stopped after 5 steps
Navigating...Fail: Episode stopped after 5 steps
```

### IV.b *Mean-field approximation,* $Q=Q(q)$, $q$-dependency respected in gradients¶

Here, the update step for $q(S\_\tau|\mathbf{A}\_{t:\tau-1})$ for $\tau>t$ is done using gradient descent, since there is no closed-form solution when the $q$-dependency of $Q$ is taken into account.

#### for $t=0,...,T-1$:¶

(1) observe $X\_t = x\_t$

(2) initialize $q(S\_0),\dots,q(S\_t),q(S\_{t+1}|A\_t),\dots,q(S\_T|A\_t,\dots,A\_{T-1})$, and $q(A\_t,\dots,A\_{T-1})$.

(3) repeat until convergence:

$$
q(S\_0) \leftarrow \textit{same as IV.a}
$$

for $\tau=1,\dots,T$:

$$
\qquad q(S\_\tau|\mathbf{A}\_{t:\tau-1}) \leftarrow \left\{ \begin{array}{cc} \textit{same as IV.a} & \text{if } \tau<t \\
\textit{same as IV.a} & \text{if } \tau = t \\
\mathrm{argmin}\_{q(S\_\tau|\mathbf{A}\_{t:\tau-1})} \underbrace{\left\langle \log \frac{q(S\_\tau|\mathbf{A}\_f) \sum\_{s\_\tau} p\_0(X\_\tau|s\_\tau) q(s\_\tau|\mathbf{A}\_f)}{\mathrm{Tr}\_{S\_\tau S\_{\tau-1}}^{A\_{\tau-1}} \mathrm{Tr}\_{S\_{\tau+1}S\_\tau}^{A\_\tau} \, p\_0(X\_\tau|S\_\tau) \, p\_\mathrm{des}(X\_\tau)} \right\rangle\_q}\_{=\langle \mathcal F\_{\mathbf{S}}(\mathbf{x}\_t,\mathbf{a}\_{t-1},\mathbf{A}\_f) - Q(q(S\_\tau|\mathbf{A}\_f))\rangle\_{q(\mathbf{A}\_f)}\,+\,\text{const.}} & \text{if } \tau>t\end{array}\right.
$$

end for

$$
q(A\_t,\dots,A\_{T-1}) \leftarrow \textit{same as IV.a}
$$

(4) Sample $a\_t$ from $\sum\_{a\_{t+1},\dots,a\_{T-1}} q(A\_t,a\_{t+1},\dots,a\_{T-1})$.

In [9]:

```
# show sample behavior↔
```

Out[9]:

In [ ]:

```
# set up grid world (jupyter notebook only)↔
```

In [22]:

```
# run the agent (jupyter notebook only)↔
```

```
Navigating...Fail: Episode stopped after 5 steps
Navigating...Fail: Episode stopped after 5 steps
Navigating...Fail: Episode stopped after 5 steps
```

### Remark: *Gridworld without inner walls*¶

The mean-field approach IV.a can succeed sometimes in simple situations such as when the inner walls in the above gridworld environment are removed:

In [6]:

```
# load trajectories from file and show most probable ones↔
```

Note that, **if the $q$-dependency of $Q$ is respected** in the update equations (IV.b) then the **resulting behavior is very different** to when it is ignored (IV.a).

### IV.c *Soft-max of* $Q = Q\_{exact} := Q(p\_\mathrm{pred})$¶

The value-function $Q$ in Active Inference has a built-in entropy term that penalizes states with bad vision (state-observation probability with high entropy) and thus the *presupposed* action distribution $\frac{1}{Z}p\_0(\mathbf{A})e^{Q(\mathbf{A})}$ produces a similar behavior as the utility-based approach, when we use the exact predictive distribution

$$
p\_\mathrm{pred}(S\_\tau|\mathbf{A}\_{t:\tau-1}) = \sum\_{s\_t,\dots,s\_{\tau-1}} \mathrm{Tr}\_{S\_\tau s\_{\tau-1}}^{A\_{\tau-1}} \cdots \mathrm{Tr}\_{s\_{t+1} s\_t}^{A\_t} \underbrace{p(s\_t|\mathbf{x}\_t,\mathbf{a}\_{t-1})}\_{\text{ exact Bayesian inference, as in I}}
$$

instead of $q(S\_\tau|\mathbf{A}\_{t:\tau-1})$ to define the value function

$$
Q\_\mathrm{exact}:=Q(p\_\mathrm{pred}) = \sum\_{\tau=t+1}^T \left\langle \log \frac{p\_0(X\_\tau|S\_\tau) p\_\mathrm{des}(X\_\tau)}{\sum\_{s\_\tau}p\_0(X\_\tau|s\_\tau) \, \color{red}{p\_\mathrm{pred}(s\_\tau|\mathbf{A}\_{t:\tau-1})}}\right\rangle\_{p\_0(X\_\tau|S\_\tau) \color{red}{p\_\mathrm{pred}(S\_\tau|\mathbf{A}\_{t:\tau-1})}}
$$

#### for $t=0,\dots,T-1$:¶

(1) observe $X\_t=x\_t$

(2) Inference over states: Same as in I.

(3) calculate $Q$ and action policy:

$$
p(\mathbf{A}\_{t:T-1}) \leftarrow \frac{1}{\text{norm.}} \, p\_0(\mathbf{A}\_{0:T-1}) e^{\alpha \sum\_{\tau=t+1}^T Q\_\tau(\mathbf{A}\_{t:\tau-1})}
$$

where $Q\_\tau(\mathbf{A}\_{t:\tau-1})$ is determined by

$$
\langle \log p\_0(X\_\tau|S\_\tau)\rangle\_{p\_0(X\_\tau|S\_\tau)p(S\_\tau|\mathbf{A}\_{t:\tau-1})}+ \langle \log p\_\mathrm{des}(X\_\tau)\rangle\_{p\_0(X\_\tau|S\_\tau)p(S\_\tau|\mathbf{A}\_{t:\tau-1})} - \left\langle \log \sum\_{s\_\tau} p\_0(X\_\tau|s\_\tau) p(s\_\tau|\mathbf{A}\_{t:\tau-1})\right\rangle\_{p\_0(X\_\tau|S\_\tau) p(S\_\tau|\mathbf{A}\_{t:\tau-1})}
$$$$
\qquad p(S\_\tau|\mathbf{A}\_{t:\tau-1}) = \sum\_{s\_t,\dots,s\_{\tau-1}} \mathrm{Tr}\_{S\_\tau s\_{\tau-1}}^{A\_{\tau-1}} \cdots \mathrm{Tr}\_{s\_{t+1} s\_t}^{A\_t} p(s\_t|\mathbf{x}\_t,\mathbf{a}\_{t-1})
$$

(4) Sample $a\_t$ from $\sum\_{a\_{t+1},\dots,a\_{T-1}} p(A\_t,a\_{t+1},\dots,a\_{T-1})$.

In [10]:

```
# show sample behavior↔
```

Out[10]:

In [ ]:

```
# set up grid world (jupyter notebook only)↔
```

In [47]:

```
# run the agent (jupyter notebook only)↔
```

```
Navigating...Done: Success after 5 steps!
Navigating...Done: Success after 5 steps!
Navigating...Done: Success after 5 steps!
```

---

## V. Direct Active Inference (Schöbel et al. 2018): *A special case of Control as Inference*¶

### V.a *Mean-field assumption*¶

#### for $t=0,...,T-1$:¶

(1) observe $X\_t = x\_t$

(2) initialize $q(S\_0),\dots,q(S\_t),q(S\_{t+1}|A\_t),\dots,q(S\_T|A\_t,\dots,A\_{T-1})$, and $q(A\_t,\dots,A\_{T-1})$.

(3) repeat until convergence:

$$
q(S\_0) \leftarrow \textit{same as IV.a}
$$

for $\tau=1,\dots,T-1$:

$$
q(S\_\tau|\mathbf{A}\_{t:\tau-1}) \leftarrow \left\{ \begin{array}{cc} \text{same as in 2016/2017} & \text{if } \tau\leq t \\
\frac{1}{\text{norm.}} \,e^{\langle \log \mathrm{Tr}\_{S\_\tau S\_{\tau-1}}^{A\_{\tau-1}}\rangle\_{q(S\_{\tau-1}|A\_t,\dots,A\_{\tau-2})} \, + \langle \log \mathrm{Tr}\_{S\_{\tau+1}S\_{\tau}}^{A\_{\tau}}\rangle\_{q(S\_{\tau+1}|A\_t,\dots,A\_{\tau})q(A\_{\tau})} \, + \langle \log p\_\mathrm{des}(X\_\tau)\rangle\_{p\_0(X\_\tau|S\_\tau)}} & \text{if } \tau>t\end{array}\right.
$$

end for

$$
q(S\_T|\mathbf{A}\_{t:T-1}) \leftarrow \frac{1}{\text{norm.}} \, e^{\langle \log \mathrm{Tr}\_{S\_T S\_{T-1}}^{A\_{T-1}}\rangle\_{q(S\_{T-1}|A\_t,\dots,A\_{T-2})} \, + \langle \log p\_\mathrm{des}(X\_T)\rangle\_{p\_0(X\_T|S\_T)}}
$$$$
q(A\_t,\dots,A\_{T-1}) \leftarrow \frac{1}{\text{norm.}}\, p\_0(\mathbf{A}\_{t:T-1}) \, e^{\alpha \sum\_{\tau={t+1}}^T G\_\tau(\mathbf{A}\_{t:T-1})}
$$

where $G\_\tau(\mathbf{A}\_{t:T-1}) = -\mathcal F\_{\mathbf{S}}(\mathbf{A}\_{t:T-1}) - D(\mathbf{A}\_{t:T-1})$ is determined by

$$
\langle \log \mathrm{Tr}\_{S\_\tau,S\_{\tau-1}}^{A\_{\tau-1}} \rangle\_{q(S\_\tau|\mathbf{A}\_{t:\tau-1}) q(S\_{\tau-1}|\mathbf{A}\_{t:\tau-2})} - \langle \log q(S\_\tau|\mathbf{A}\_{t:\tau-1})\rangle\_{q(S\_\tau|\mathbf{A}\_{t:\tau-1})} + \langle \log p\_\mathrm{des}(X\_\tau)\rangle\_{p\_0(X\_\tau|S\_\tau) q(S\_\tau|\mathbf{A}\_{t:\tau-1})}
$$

(4) Sample $a\_t$ from $\sum\_{a\_{t+1},\dots,a\_{T-1}} q(A\_t,a\_{t+1},\dots,a\_{T-1})$.

In [12]:

```
# show sample behavior↔
```

Out[12]:

In [ ]:

```
# set up grid world (jupyter notebook only)↔
```

In [7]:

```
# run the agent (jupyter notebook only)↔
```

```
Navigating...Fail: Episode stopped after 5 steps
Navigating...Fail: Episode stopped after 5 steps
Navigating...Fail: Episode stopped after 5 steps
```

### V.b *Bethe assumption*¶

Since the Bethe assumption leads to a belief propagation algorithm that results in exact factor marginals, the resulting behavior is identical with Control as Inference (Section II above). In S1 Appendix we provide more details and a derivation of the update equations for the one-step example from the article.

---

# 2. *Explanation*¶

The action policies in the above algorithms are essentially given by (for simplicity neglecting fixed action priors and considering only one step)

$$
p(A) = \left\{\begin{array}{cc} \sum\_{x}\underbrace{\frac{p\_\mathrm{pred}(x|A)}{\mathcal Z(x)}}\_{=p\_\mathrm{Bayes}(A|x)} \, p\_\mathrm{des}(x) & (\textit{Conditioning on $p\_\mathrm{des}$}) \\ \frac{1}{\mathcal Z}\, \sum\_{x} p\_\mathrm{pred}(x|A) \, p\_\mathrm{des}(x) & (\textit{Conditioning on success}) \\ \frac{1}{\mathcal Z} \, \exp\Big[ \sum\_x p\_\mathrm{pred}(x|A) \, \log p\_\mathrm{des}(x)\Big] & (\textit{Expected Utility}) \\
\frac{1}{\mathcal Z} \exp\Big[\underbrace{\sum\_x q(x|A) \, \log p\_\mathrm{des}(x) + \textit{other terms}}\_{= \,-F\_S(A)+Q(q)} \Big] & (\textit{$Q$-value Active Inference}) \\
\frac{1}{\mathcal Z} \exp\Big[ \underbrace{-D\_\mathrm{KL}(p\_\mathrm{pred}(X|A)\|p\_\mathrm{des}(X)) - \langle H(p\_0(X|S))\rangle\_{p\_0(X,S|A)}}\_{= \, Q(p\_\mathrm{pred})\, =\, \langle \log p\_\mathrm{des} \rangle + \langle D\_\mathrm{KL}(p\_0(X|S)\|p\_\mathrm{pred}(X|A))\rangle }\Big] & (\textit{softmax of $Q\_\mathrm{exact}=Q(p\_\mathrm{pred})$})\end{array}\right.
$$

where $p\_\mathrm{pred}(X|A)$ denotes the predictive distribution of future consequences $X$ given the action $A$ resulting from the generative model $p\_0$, $\mathcal Z$ denotes normalization constants, and $\mathcal Z(x)=\sum\_a p\_\mathrm{pred}(x|a)$ normalizes the Bayes posterior $p(A|x)$ in Jeffrey's conditioning rule.

We can now understand the behaviour of the algorithms as follows:

- **Conditioning on $p\_\mathrm{des}$ vs. conditioning on success**: When comparing the two ways of directly performing inference over actions, i.e. Jeffrey conditioning and Control as Inference (e.g. Toussaint 2006), we can see that they only differ in the order of normalizing and taking the expected value over $X$. This means that, in the approach using naive Jeffrey conditionalization, first a goal $X=x$ is sampled from $p\_\mathrm{des}$ and then a path is planned by calculating $p(A|x)$, which is why the simulation can fail in case the top goal is sampled from $p\_\mathrm{des}$, which is in an area of bad vision. In contrast, Toussaint's version of inference weighs the desirability of an outcome $p\_\mathrm{des}(x)$ with its realizability given by $p\_\mathrm{pred}(x|A)$, so that which $x$ is chosen to plan a path depends on how well it can be navigated to.
- **Expected Utility**: Similar to Control as Inference, in expected utility approaches the utility of outcomes, e.g. $U(x)=\log p\_\mathrm{des}(x)$, is weighted with their realizability $p\_\mathrm{pred}(x|A)$ to decide which $A$'s should be preferred. The difference to Control as Inference is how the desired distribution is transformed into a utility, which here is a completely arbitrary choice (for comparison's sake, we assume a desired distribution $p\_\mathrm{des}$ for all approaches, which is uniformly distributed over the goal states).
- **Q-value Active Inference**: In $Q$-value Active Inference based on mean-field approximations, the action distribution contains contributions from the state updates (perception steps), due to the shared variational free energy as an optimization objective. However, there are two issues here:

  - The mean-field assumption seems to be **too restrictive** for reliable navigation in a grid world (we encourage the reader to play around with other grid world setups in the jupyter notebook on github).
  - Neglecting the $q$-dependency of $Q$, as is done in the vast majority of Active Inference literature, **produces different behavior** than when this dependency is included using a gradient descent algorithm. This is a fundamental issue with $Q$-value active inference that is nowhere acknowledged in the literature (it is instead considered of little importance for numerical simulations in Friston et al. 2016), even though in our grid world simulation the inclusion of this dependency produces unexplainable behavior (see IV.b above). Instead, one usually motivates the exclusion of this $q$-dependency by a separation of the full free energy adopted from variational Bayesian inference with fixed references, where it follows from the fact that the full free energy can be written as a sum of a free energy over states and a KL divergence that is independent of state distributions because there the reference does not have the extra $q$-dependency (see S4 Appendix for details). However, for Active Inference this essentially means that one gives up the optimization of a single free energy but optimizes two different free energy expressions for action and perception.

- **Softmax of $Q\_\mathrm{exact}$**: When using the true predictive distribution $p\_\mathrm{pred}(S|A)$ instead of the trial distribution $q(S|A)$ in $Q$, then the resulting behavior from a soft-max action distribution is similar to Control as Inference and the expected utility approach. This is partly because $Q$ has a built-in term that explicitly punishes high outcome variability (high entropy of $p\_0(X|S)$), resulting in a bias towards the bottom target, but also because $Q\_\mathrm{exact}$ differs from the expected utility $\langle \log p\_\mathrm{des}\rangle$ only by the average KL divergence between $p\_0(X|S)$ and $p\_\mathrm{pred}(X|A)$, which is small for a perfect transition matrix. Note that the extra dependency of $Q$ on $p\_0(X|S)$ can, however, introduce a strong behavioral variability, in the sense that it can result in qualitatively different behavior in situations where $p\_\mathrm{pred}(X|A)$ is constant (see the simple example in Fig 8 of the article).

In [ ]:

```

```
